# A functional connection between translation elongation and protein folding at the ribosome exit tunnel in *Saccharomyces cerevisiae*

**DOI:** 10.1093/nar/gkaa1200

**Published:** 2020-12-16

**Authors:** Olga Rodríguez-Galán, Juan J García-Gómez, Iván V Rosado, Wu Wei, Alfonso Méndez-Godoy, Benjamin Pillet, Alisa Alekseenko, Lars M Steinmetz, Vicent Pelechano, Dieter Kressler, Jesús de la Cruz

**Affiliations:** Instituto de Biomedicina de Sevilla (IBiS), Hospital Universitario Virgen del Rocío/CSIC/Universidad de Sevilla, Seville, Spain; Departamento de Genética, Universidad de Sevilla, Seville, Spain; Instituto de Biomedicina de Sevilla (IBiS), Hospital Universitario Virgen del Rocío/CSIC/Universidad de Sevilla, Seville, Spain; Departamento de Genética, Universidad de Sevilla, Seville, Spain; Instituto de Biomedicina de Sevilla (IBiS), Hospital Universitario Virgen del Rocío/CSIC/Universidad de Sevilla, Seville, Spain; Departamento de Genética, Universidad de Sevilla, Seville, Spain; Stanford Genome Technology Center, Stanford University, Palo Alto, CA, USA; CAS Key Lab of Computational Biology, CAS-MPG Partner Institute for Computational Biology, Shanghai Institute of Nutrition and Health, Shanghai Institutes for Biological Sciences, University of Chinese Academy of Sciences, Chinese Academy of Sciences, Shanghai, China; Unit of Biochemistry, Department of Biology, University of Fribourg, Fribourg, Switzerland; Unit of Biochemistry, Department of Biology, University of Fribourg, Fribourg, Switzerland; SciLifeLab, Department of Microbiology, Tumor and Cell Biology. Karolinska Institutet, Solna, Sweden; Stanford Genome Technology Center, Stanford University, Palo Alto, CA, USA; European Molecular Biology Laboratory (EMBL), Genome Biology Unit, Heidelberg, Germany; Department of Genetics, School of Medicine, Stanford, CA, USA; SciLifeLab, Department of Microbiology, Tumor and Cell Biology. Karolinska Institutet, Solna, Sweden; Unit of Biochemistry, Department of Biology, University of Fribourg, Fribourg, Switzerland; Instituto de Biomedicina de Sevilla (IBiS), Hospital Universitario Virgen del Rocío/CSIC/Universidad de Sevilla, Seville, Spain; Departamento de Genética, Universidad de Sevilla, Seville, Spain

## Abstract

Proteostasis needs to be tightly controlled to meet the cellular demand for correctly *de novo* folded proteins and to avoid protein aggregation. While a coupling between translation rate and co-translational folding, likely involving an interplay between the ribosome and its associated chaperones, clearly appears to exist, the underlying mechanisms and the contribution of ribosomal proteins remain to be explored. The ribosomal protein uL3 contains a long internal loop whose tip region is in close proximity to the ribosomal peptidyl transferase center. Intriguingly, the *rpl3*[W255C] allele, in which the residue making the closest contact to this catalytic site is mutated, affects diverse aspects of ribosome biogenesis and function. Here, we have uncovered, by performing a synthetic lethal screen with this allele, an unexpected link between translation and the folding of nascent proteins by the ribosome-associated Ssb-RAC chaperone system. Our results reveal that uL3 and Ssb-RAC cooperate to prevent 80S ribosomes from piling up within the 5′ region of mRNAs early on during translation elongation. Together, our study provides compelling *in vivo* evidence for a functional connection between peptide bond formation at the peptidyl transferase center and chaperone-assisted *de novo* folding of nascent polypeptides at the solvent-side of the peptide exit tunnel.

## INTRODUCTION

Protein homeostasis (proteostasis) is controlled by a network of cellular mechanisms that ensures the optimal concentration and composition of correctly folded proteins within cells under normal conditions. Key components of this network are the molecular chaperones and the cellular protein degradation machineries. The different molecular chaperones act in diverse manners on proteins, they assist the folding of nascent proteins (*de novo* folding), participate in the refolding of stress-denatured proteins, promote the assembly of oligomeric protein complexes, mediate protein degradation and prevent formation or promote disassembly of protein aggregates (1–3). An intact proteostasis network is strictly required to protect cells from the detrimental effects of a variety of internal and external perturbations. Importantly, loss of proteostasis maintenance is an underlying cause of aging and numerous diseases associated with the accumulation and aggregation of misfolded proteins (4,5).

In eukaryotes, molecular chaperones involved in the *de novo* folding of cytosolic proteins can be divided into two groups based on their ability to interact with ribosomes and the nascent polypeptides. The first group includes two distinct ribosome-associated chaperone systems that bind in close proximity to the exit site of the ribosomal peptide exit tunnel (PET), the α•β-heterodimeric nascent polypeptide-associated complex (NAC) and the Hsp70•Hsp40-based ribosome-associated complex (RAC), which is part of the so-called chaperone triad or Ssb-RAC complex (4,6,7). In the yeast *Saccharomyces cerevisiae*, NAC is comprised by the α-subunit Egd2 and either one of two β-subunits, Egd1 or Btt1 (reviewed in (7,8)). In turn, RAC is a stable heterodimeric complex, composed of the catalytically-inactive Hsp70 protein Ssz1 and the Hsp40 protein Zuo1 (Zuotin), that binds to the Hsp70 paralogue Ssb1 or Ssb2 to form the chaperone triad (3,6,9). The second group comprises different systems of cytosolic chaperones that appear to act downstream of the ribosome-associated NAC and RAC, such as prefoldin, the Hsp70•Hsp40 dimer, and the group II chaperonin TRiC/CCT complex (reviewed in (3,10–12)). Prefoldin is a heterohexameric complex that in yeast is composed of the non-essential Pfd1/Gim6, Pfd2/Gim4, Pfd3/Gim2/Pac10, Pfd4/Gim3, Pfd5/Gim5 and Pdf6/Gim1/Yke2 subunits ((13), reviewed in (14)). The Hsp70•Hsp40 dimer is composed of an Hsp70 ATPase of the Ssa family (Ssa1, Ssa2, Ssa3 or Ssa4) and a J-domain containing Hsp40 co-chaperone (Ydj1 or Sis1) (15,16). Finally, the chaperonin TRiC/CCT is a large complex consisting of two back-to-back stacked rings enclosing a central folding cavity; each ring is formed by eight different paralogous subunits, named Cct1 to Cct8 in yeast (17). In addition to these general systems, the fail-safe production of certain ribosomal proteins (r-proteins) is co-translationally assisted by specific protein factors, referred to as dedicated chaperones of r-proteins (18–20).

The following substrate specificities have been uncovered for some of these different chaperone systems in yeast. NAC appears to interact with different proteins depending on which β-subunit is present in the complex: (i) NAC Egd1/Egd2 associates with ribosomes translating mRNAs encoding metabolic enzymes and endoplasmic reticulum (ER)-targeted secretory/membrane proteins, with the latter also being recognized by the signal recognition particle (SRP); (ii) NAC Btt1/Egd2 associates with ribosomes translating cytosolic r-proteins and nuclear-encoded mitochondrial proteins (21). In turn, the Ssb-RAC chaperone triad binds to nascent polypeptides of >80% of all cytoplasmic and nuclear proteins, but also to about 80% of nascent mitochondrial proteins and 40% of the ER-targeted proteins (22,23). All these proteins are generally long, multi-domain, and aggregation-prone molecules that exhibit hydrophobic patches and intrinsically disordered regions (22). This broad specificity is consistent with the fact that deletion of *SSB1* and *SSB2* or either of the two genes encoding the RAC subunits (Zuo1 or Ssz1) leads to widespread aggregation of nascent proteins, including many r-proteins (22,24). Prefoldin and TRiC/CCT recognize a more restricted range of proteins (about 5% of all cytosolic proteins) (25). Nascent cytoskeletal proteins are the best-known substrates of prefoldin, which captures unfolded actin and α-/ß-tubulin co-translationally and delivers them to TRiC/CCT for folding (13,25).

Chaperones involved in the *de novo* folding of proteins have been described as modulators of protein synthesis; thus, it has been shown that cellular insults that induce the misfolding of nascent proteins can attenuate the rate of protein synthesis *via* pausing the elongation step of translation (e.g. (26,27)). In turn, the speed of translation can also affect the folding efficiency of proteins; slowing down translation improves protein folding, while increasing the elongation rate reduces the protein folding efficiency (reviewed in (10,11,28,29)). Here, we report on a striking functional link between translation and *de novo* folding of proteins in yeast that we uncovered by performing a synthetic lethal screen with the *rpl3*[W255C] allele. The W255 residue of r-protein uL3 (formerly r-protein L3) makes the closest approach of any amino acid to the ribosomal peptidyl transferase center (PTC), which is partially distorted at its A-site tRNA-binding pocket by the W255C mutation (30). Previous results have shown that the *rpl3*[W255C] mutation affects ribosome function in a diverse manner: it confers resistance to peptidyl-transferase antibiotics such as trichodermin and anisomycin (31,32), promotes the inability of cells to maintain the killer virus (33), and results in reduced translation, increased affinity for aminoacyl-tRNAs, decreased peptidyl-transferase activity, decreased affinity for eukaryotic elongation factor 2 (eEF2), and increased efficiency of programmed –1 ribosomal frameshifting (see (34) and references therein). Notably, this mutation also impairs cytoplasmic maturation of pre-40S ribosomal subunits (r-subunits) by preventing the stimulation of the GTPase activity of eIF5B/Fun12, which is required for the Nob1-dependent cleavage of the 20S precursor rRNA (pre-rRNA) at site D to produce the mature 18S rRNA 3′ end (for details on the yeast pre-rRNA processing pathway, see Supplementary Figure S1 and its legend) (35,36). Herein, we reveal a specific synthetic lethal (sl) interaction between the *rpl3*[W255C] allele and null mutants of the ribosome-associated Ssb-RAC and the cytosolic Hsp70•Hsp40 chaperone systems. Our results indicate that this synthetic lethality is not simply due to an enhancement of the ribosome biogenesis defect caused by the *rpl3*[W255C] mutation or a significant augmentation of the aggregation propensity associated with the chaperone mutations, but appears to be related to an impairment of early translation elongation. We show that the absence of RAC clearly causes an enhancement of the tendency of uL3[W255C]-containing ribosomes to pile up within the 5′ region of mRNAs. Taken together, our analysis provides compelling *in vivo* evidence for a functional link between peptide bond formation at the PTC and co-translational chaperoning of nascent polypeptides at the exit site of the PET.

## MATERIALS AND METHODS

### Yeast strains, plasmids, and microbiological methods

All *S. cerevisiae* strains used in this study are listed in Supplementary Table S1. Unless otherwise indicated, experiments were conducted in the W303 genetic background. YDK145-7D was used as the sl-screen starting strain. Growth and handling of yeast as well as standard media (YPD, YPGal, SD and SGal media) were done according to established procedures (37). Deletion disruptions with the heterologous kanMX4 or HIS3MX6 marker modules were carried out as previously described (38,39). Yeast cells were transformed by a lithium acetate method (40). Tetrads were dissected with a MSM200 micromanipulator (Singer Instruments, UK). Plasmids used in this study are listed in Supplementary Table S2. Recombinant DNA techniques were done according to established procedures using *Escherichia coli* DH5α for cloning and plasmid propagation (41). Inserts of all plasmid constructs were fully sequenced. More information on the plasmids is available on request.

### Synthetic lethal screen and cloning of *ZUO1*

The strain YDK145-7D bearing the *rpl3*[W255C] allele on a centromeric plasmid containing the *TRP1* marker was screened for sl mutations based on a combination of the *ade2 ade3* red/white colony-sectoring assay with the counter-selection of Ura^+^ cells on 5-FOA-containing plates (42). This strain was grown in liquid SD-Trp-Ura medium to an optical density at 600 nm (OD_600_) of around 0.5 and plated on SD-Trp plates at a density of ca. 500 cells/plate. The plates were then UV irradiated, resulting in ca. 15–30% survival, and incubated for 5 days at 30°C in the dark. About 45000 independent colonies were screened. Red colonies were chosen and restreaked once on SD-Trp plates and then twice on SD-Trp and 5-FOA-containing plates. Non-sectoring red and 5-FOA-sensitive colonies were then selected as sl candidates. To confirm that both phenotypes were neither due to genomic integration of the plasmid pHT4467Δ-RPL3 nor linked to a complete loss-of-function mutation of the *rpl3*[W255C] allele, the sl candidates were transformed with either YCplac111-RPL3 or YCplac111-rpl3[W255C]; true sl candidates restored sectoring and growth on 5-FOA-containing plates only upon transformation with YCplac111-RPL3. Nine strains showed a clear synthetic enhancement (se) or an sl phenotype, but only one of them (sl7D30/2; JDY457 strain) was chosen for further analysis due to its net deficit in 40S r-subunits and strong slow-growth (sg) phenotype at 30°C. This candidate was transformed with a YCplac111-based yeast genomic library (38,43) and screened for clones complementing its sg phenotype on SD-Leu-Trp plates at 30°C. Candidate plasmids were isolated from yeast, amplified in *E. coli*, and re-transformed into JDY457. Sequence analysis revealed an insert of ca. 8 kb, including *ZUO1*, *ERV29* and *YGR283C*. Further subcloning indicated that the presence of the *ZUO1* gene was sufficient to complement the sl phenotype and other distinct features (i.e. aberrant polysome profile) of the sl7D30/2 candidate. PCR amplification and sequencing revealed that the original *zuo1* mutation of sl7D30/2 was an A-to-T conversion at nucleotide position 676, which changes the arginine codon (AGA) at amino acid position 226 to a premature stop codon (TGA).

### Synthetic interaction crosses

To determine whether mutant alleles affecting genes coding for chaperones linked to protein synthesis were synthetically lethal with *rpl3* alleles, selective crosses were performed between chaperones or chaperone-cofactor mutants and a *RPL3* shuffle strain (either YDK39-1D or YDK39-1C), the resulting diploids were sporulated and tetrads dissected. Spore clones with the appropriate markers were selected and transformed with YCplac111-borne *RPL3* alleles. Transformants were restreaked on SD-Leu plates and subjected to plasmid shuffling on 5-FOA-contaning plates. More information on the different crosses is available on request.

### Polysome analysis

Cell extracts for polysome analyses were prepared and analyzed as previously described (44,45). Ten *A*_260_ units of cell extracts were loaded onto sucrose gradients. Profiles were obtained by continuously monitoring the *A*_254_ with a Teledyne-ISCO UA-6 system.

### Analysis of newly synthesized and aggregated proteins

Newly synthesized uL3-2xHA protein, expressed for 20 min from a copper-inducible promoter, was induced and analyzed as exactly described in Pillet *et al.* (46). Isolation of aggregated proteins was done from cellular lysates as exactly described in the procedure established by Koplin *et al.* (24), except for the sonication modifications described in (47). Whole cell extracts and aggregated protein samples were resuspended in SDS sample buffer, separated by SDS-PAGE using TGX^TM^ precast gels (Bio-Rad), and analyzed by colloidal blue Coomassie staining. The lane profiles of the separated proteins loaded in each well were obtained using the Image Lab software provided for the ChemiDoc MP imaging system and the relative total protein intensity value was calculated for each profile.

### Western blot analysis

Western blot analysis was performed according to standard procedures using the following primary antibodies: mouse monoclonal anti-HA 16B12 (Covance) and anti-uL3 (48) and rabbit polyclonal anti-uL1 (49), anti-uL29 (50), anti-Adh1 (provided by the De Virgilio laboratory; University of Fribourg), and anti-Kar2 (gift of R. Schekman). Secondary goat anti-mouse or anti-rabbit horseradish peroxidase-conjugated antibodies (Bio-Rad) were used. Proteins were detected using a chemiluminescence detection kit (Super-Signal West Pico, Pierce) and a ChemiDoc™ MP system (Bio-Rad).

### RNA analysis

RNA was extracted from yeast cells using the hot phenol procedure (37). The amount of RNA was quantified in a Nanodrop 2000 Spectrophotometer (Thermo Scientific) and RNA integrity was checked by agarose gel electrophoresis. Northern hybridization analyses were carried out according to standard procedures (51). In all experiments, RNA was extracted from samples corresponding to 10 OD_600_ units of exponentially grown cells. Equal amounts of total RNA (5 μg) were loaded on 1.2% agarose-6% formaldehyde or on 7% polyacrylamide-8M urea gels (51). Specific oligonucleotides were 5′-end labeled with [γ-32P] ATP and used as probes. The sequences of oligonucleotides used for northern hybridization are listed in Supplementary Table S3. Their hybridization positions in the pre-rRNA are shown in Supplementary Figure S1. Phosphorimager analysis was performed with a Typhoon™FLA9400 imaging system (GE Healthcare) and quantified using the GelQuant.NET software (biochemlabsolutions.com).

### 5′P sequencing analyses

The 5′P sequencing (5PSeq) method was performed as previously described (52,53) using 100 μg total RNA, isolated from the different strains, grown to exponential phase in SGal medium and shifted to SD medium for 18 h, as starting material. In brief, an RNA oligonucleotide (rP5_RND) containing an Illumina adaptor and unique molecular identifiers (UMI) was ligated to the intermediates of co-translational 5′-3′ mRNA degradation (5′P). Partially polyadenylated molecules were enriched using Dynabeads oligo (dT)_25_ and used for the generation of Illumina-compatible libraries. Samples were multiplexed and sequenced in a HiSeq 2000 lane. 5′-ends of reads were mapped and PCR duplicates collapsed using UMI information. Experiments were performed in biological duplicates. Raw and processed sequencing data were deposited at Gene Expression Omnibus (GEO) with accession number GSE114899. Additional 5PSeq analyses were performed using the HT-5PSeq approach (54), which increases the coverage both at the 5′ and 3′ regions in comparison with the oligo-dT enriched 5PSeq. Experiments were performed in biological triplicates and 6 μg total RNA was used as input per strain and replicate. Both random hexamer and oligo-dT primers were used in reverse transcription, after which ribosomal cDNA was depleted using duplex-specific nuclease and oligonucleotide probes. Resulting libraries were sequenced on a Nextseq 500. Data was analyzed using the Fivepseq package (55). Raw and processed sequencing data were deposited at GEO with accession number GSE151632. 5PSeq data was visualized as metagenes normalized by mapped library sized around the start and stop codon in reads per million (sum rpm). To decrease the effect of outliers, in 5PSeq extreme values (top 5%) have been flattened down to 95%. For HT-5PSeq we used the default outlier detection as implemented in the Fivepseq package (55). Interactive browsable files can be accessed via the SciLifeLab data repository (https://figshare.com/s/9e695e6f6c1b97cd79ce). To measure the relative accumulation of ribosome protection in the 5′ region of the genes, we compared the relative 5PSeq coverage of the mRNA regions encoding the first *versus* the last 100 amino acids (Log_2_ 5′/3′). Only genes whose mRNAs were long enough to encode 200 amino acids and covered by at least 10 5PSeq reads in each region have been considered (1118 genes in total). Significance of the mean ranks difference was assessed using a Wilcoxon paired signed-rank test.

### Statistical significance

All experiments were performed in duplicate or triplicate (biological replicates), normally each with at least two technical replicates to give a sum of at least four or six recordings respectively. In most figures, only a representative result is shown. In distinct experiments, the results represent the mean and the standard deviation values. When used, *P*-values less than 0.05 were considered statistically significant.

## RESULTS

### Synthetic lethality connects the *rpl3*[W255C] allele with the Ssb-RAC chaperone triad

The *rpl3*[W255C] is a highly pleiotropic allele that negatively affects different aspects of both ribosome biogenesis and translation. In order to get more insight into the essential function(s) of the W255 residue of uL3, we performed a sl screen with a strain harboring the *rpl3*[W255C] allele, which exhibits only a mild growth defect at 30°C (35,56); thus, enabling the isolation of nine red, non-sectoring, and 5-FOA-sensitive candidates. As described in Material and Methods, one of these candidates showed a strong sg phenotype at 30°C due to a single mutation. Complementation by a genomic library demonstrated that *ZUO1* was sufficient to restore wild-type growth, red/white sectoring, and growth on 5-FOA-containing plates of the sl-mutant. Moreover, PCR amplification and sequencing of the *zuo1* allele of the original sl-mutant strain revealed that the codon specifying arginine 226 (AGA) was mutated to a premature stop codon (TGA).

**Figure 1.**
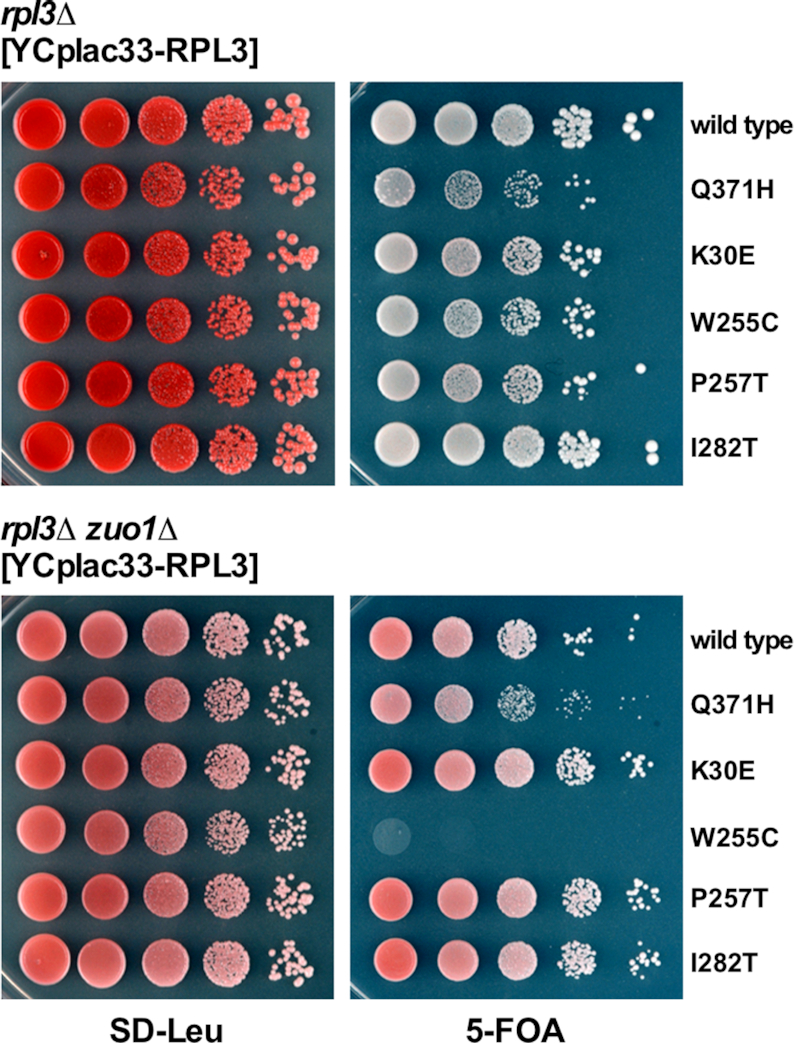
Specific synthetic lethal interaction between the *rpl3*[W255C] and the *zuo1Δ* mutant. A *rpl3Δ zuo1Δ* double mutant (YDK157-2D) and a reference *rpl3Δ* strain (YDK157-2B), both derived from the same diploid and containing wild-type *RPL3* on an *URA3* plasmid, were transformed with *LEU2* plasmids harboring either wild-type *RPL3* or the indicated *rpl3* mutant alleles. Cells were spotted in 10-fold serial dilution steps onto SD-Leu or SD + 5-FOA plates, which were incubated for 4 days at 30°C.

To independently confirm this sl-relation, we constructed a clean *RPL3 zuo1Δ* shuffle strain, which was transformed with various plasmids expressing different *rpl3* alleles. Then, growth was analyzed after plasmid shuffling on 5-FOA-containing plates. As expected, the *rpl3*[W255C] allele showed a sl-phenotype with the *zuo1Δ* null mutant (Figure 1). Interestingly, this genetic interaction was allele specific as none of the other tested *rpl3* alleles conferred synthetic lethality in combination with *zuo1Δ* (Figure 1).

**Figure 2.**
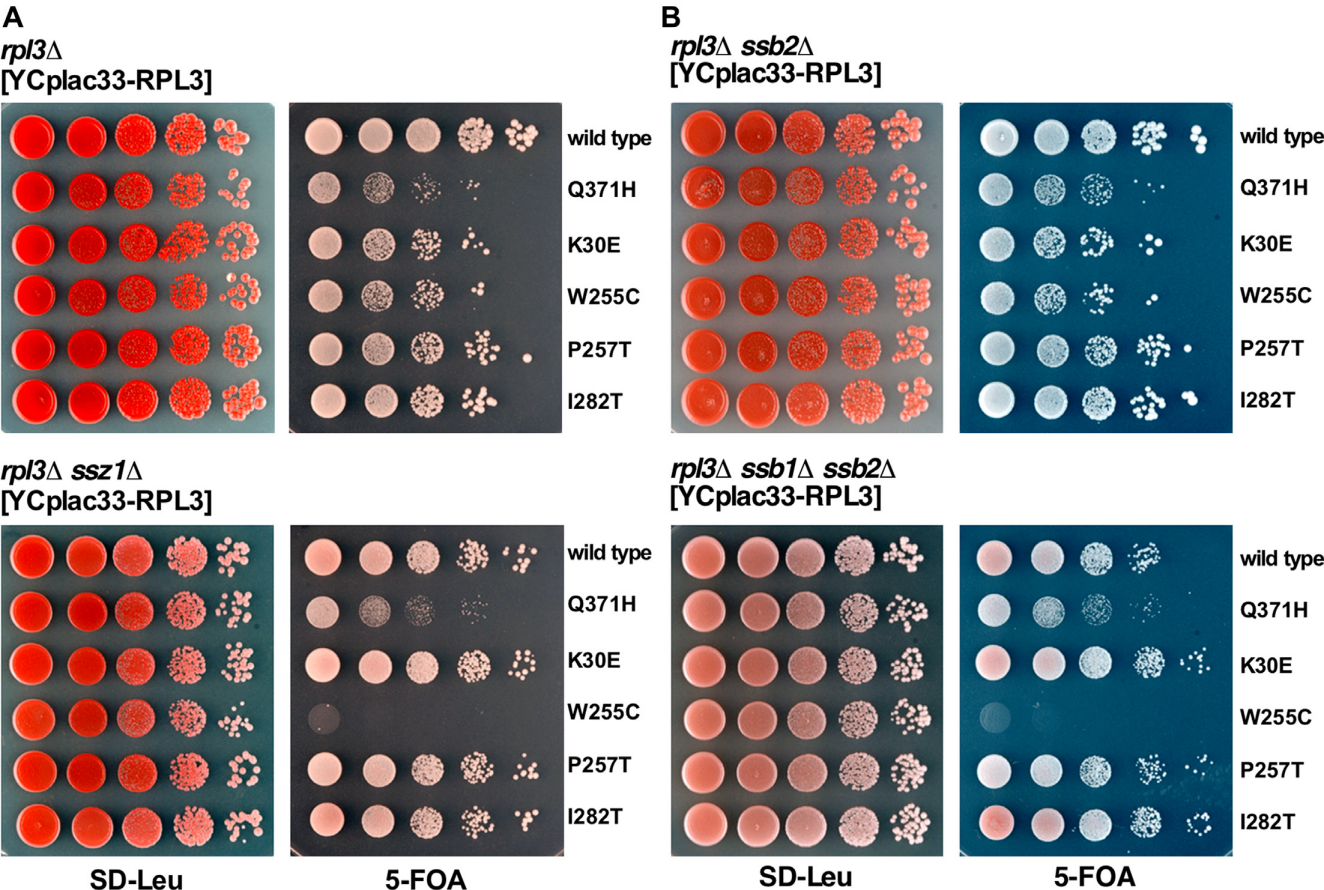
Specific synthetic lethal interaction between the *rpl3*[W255C] and the *ssz1Δ* or *ssb1Δ ssb2Δ* mutant. (**A**) A *rpl3Δ ssz1Δ* double mutant (YKD9-6C) and a reference *rpl3Δ* strain (YKD9-6B), both derived from the same diploid and containing wild-type *RPL3* on an *URA3* plasmid, and (**B**) a *rpl3Δ ssb1Δ ssb2Δ* triple mutant (YKD7-9D) and a reference *rpl3Δ ssb2Δ* double mutant strain (YKD7-9A), both derived from the same diploid and containing wild-type *RPL3* on an *URA3* plasmid, were transformed with *LEU2* plasmids harboring either wild-type *RPL3* or the indicated *rpl3* mutant alleles. Cells were spotted in 10-fold serial dilution steps onto SD-Leu or SD+5-FOA plates, which were incubated for 4 days at 30°C.

As Zuo1 is a component of the chaperone triad, which also consists of the Hsp70 proteins Ssz1 and Ssb1/2, we tested for genetic interactions between *RPL3* and *SSZ1* or *SSB1*/2. As shown in Figure 2, combining the *rpl3*[W255C] allele with either the *ssz1Δ* or the double *ssb1Δ ssb2Δ* null mutations also resulted in a sl-relation. As above, this interaction was specific for the *rpl3*[W255C] allele.

To extend the network of interactions to other chaperones involved in the *de novo* folding of proteins, we also tested for synthetic lethality between *RPL3* and *EGD2*, which encodes the α-subunit of NAC, and between *RPL3* and *GIM2*, coding for a subunit of prefoldin. As shown in Supplementary Figure S2A and B, the *egd2Δ* null mutation does not enhance the growth defect of any of the tested *rpl3* alleles, while the growth defect of the *gim2Δ* null mutation is only slightly exacerbated by the *rpl3*[W255C] allele. N^α^-acetyltransferases acetylate the N-terminal end of nascent proteins, usually co-translationally, and are positioned at the exit site of the PET (57,58). NatA, which is composed of the catalytic Ard1 and the ribosome-anchoring Nat1 and Nat5 subunits (58), is the major N^α^-acetyltransferase in yeast (59). To assess whether there is a genetic interplay with NatA, we combined the *rpl3*[W255C] allele with the *nat1Δ* mutation. As shown in Supplementary Figure S2C, no significant synthetic growth enhancement was observed in the double mutant compared to the individual single mutants; thus, suggesting a lack of functional interaction between uL3[W255C] and NatA.

Next, we also examined whether the *rpl3*[W255C] allele genetically interacts with the essential Ssa family of cytosolic Hsp70 proteins. As abovementioned, yeast encodes four functionally redundant Ssa proteins (Ssa1 to Ssa4) that appear to act downstream of the ribosome-associated chaperones, taking over folding of nascent or newly synthesized proteins ((60); reviewed in (3)). The Ssa-containing Hsp70•Hsp40 chaperones do not directly interact with ribosomes but instead with the newly translated polypeptides, although to a much smaller extent than the Ssb-RAC complex (60). While the complete absence of all four *SSA* genes causes lethality, the simultaneous deletion of *SSA1* and *SSA2* confers a sg phenotype at 30°C (61). As shown in Supplementary Figure S3, the combination of the *rpl3*[W255C] allele with the *ssa1Δ ssa2Δ* double null mutant resulted in a practically lethal phenotype.

We conclude that cells harboring the *rpl3*[W255C] mutation can no longer sustain growth in the absence of functional Ssb-RAC or when the levels of cytosolic Ssa chaperones are decreased. The allele specificity of the interaction strongly suggests an essential functional relationship between the W255-finger of uL3 at the PTC and the co-translational folding of nascent proteins mediated by the Ssb-RAC complex. The fact that the double *ssa1Δ ssa2Δ* deletion also severely compromises the growth of the *rpl3*[W255C] mutant indicates that this relationship persists even when the nascent proteins are being released from the ribosomes. In agreement with this hypothesis, *rpl3*[W255C] cells are especially sensitive to the proline analogue azetidine-2-carboxylic acid (AZC), which when incorporated into nascent proteins prevents their optimal folding (see (60)). Notably, the sensitivity is already evident at concentrations that are clearly insufficient to inhibit the growth of mutants lacking RAC components (Supplementary Figure S4), which have been previously reported to be sensitive to this drug (60).

### The uL3[W255C] mutant protein is expressed to the wild-type extent, assembled into 60S r-subunits, and engaged in translation in *zuo1Δ* cells

Although the *rpl3*[W255C] allele does not confer a severe growth defect, the replacement of a tryptophan with a cysteine in the uL3 protein could be affecting its expression, ribosomal assembly or functionality in the absence of the above studied chaperones. We therefore determined whether the observed sl-interaction could be due to a drastic reduction of the stability of the uL3[W255C] protein upon deletion of individual Ssb-RAC subunits. To this end, we expressed C-terminally 2xHA-tagged wild-type uL3, uL3[W255C] or uL3[P257T] proteins from the copper-inducible *CUP1* promoter, in either wild-type or *zuo1Δ* mutant cells, and revealed the levels of newly synthetized uL3 proteins by western blotting using an anti-HA antibody. As shown in Supplementary Figure S5, the uL3[W255C] protein was expressed at similar levels as wild-type uL3 in *zuo1Δ* cells.

Next, we tested whether the uL3[W255C] protein was efficiently incorporated into mature ribosomes and if this incorporation interfered with translation. To do so, we again expressed wild-type uL3-2xHA or mutant uL3[W255C]-2xHA from the *CUP1* promoter in wild-type and *zuo1Δ* cells and subjected cell extracts, obtained under polysome-preserving conditions (see Materials and Methods), to sucrose gradient centrifugation and fractionation. Then, the fractions were analyzed by SDS-PAGE and western blotting using both anti-HA and anti-uL3 antibodies. As shown in Supplementary Figure S6, both wild-type uL3-2xHA and uL3[W255C]-2xHA was peaking in the 60S, 80S/monosome and polysomal fractions in wild-type or *zuo1Δ* cells. Most importantly, this distribution was apparently similar to that of endogenous uL3 both in wild-type and *zuo1Δ* cells. These results indicated that uL3[W255C] gets assembled into 60S r-subunits that are efficiently recruited into polysomes, even in cells lacking the RAC complex. In support of this conclusion, increased dosage of uL3[W255C] was unable to alleviate the synthetic lethality of *rpl3*[W255C] *zuo1Δ* cells (Supplementary Figure S7A), while it clearly exerted a negative effect on the growth of *zuo1Δ* cells (Supplementary Figure S7B), suggesting that this variant efficiently competes with wild-type uL3 for incorporation into 60S r-subunits.

Altogether, these data indicate that the uL3[W255C] protein is efficiently produced and assembled into 60S r-subunits that are able to engage in translation in cells lacking the function of the RAC complex. Consequently, the sl-interaction between the *rpl3*[W255C] and *zuo1Δ* alleles, and by extension with other components of the Ssb-RAC complex, cannot be explained by a reduction of the stability and ribosomal assembly of uL3[W255C] or of the translation-competence of uL3[W255C]-containing ribosomes in these conditions.

### The genetic interaction between the *rpl3*[W255C] allele and loss-of-function mutations in Ssb-RAC cannot be rationalized by enhanced defects in ribosome biogenesis

The *rpl3*[W255C] mutation leads to a cytoplasmic 40S r-subunit biogenesis defect due to an impairment of 20S pre-rRNA processing (35,62,63). Conversely, *zuo1Δ* and *ssb1Δ ssb2Δ* cells exhibit a 60S r-subunit deficit as the result of a block in 27S pre-rRNA processing in the BY4741 genetic background (64), but a 40S r-subunit deficit in the W303 genetic background (65). We therefore wanted to determine whether the observed sl-interaction between the *rpl3*[W255C] allele and the deletion of individual Ssb-RAC subunits could be due to a synergistic impairment of the ribosome biogenesis defect of *rpl3*[W255C] cells. To this end, we first extracted total RNA from the Ssb-RAC mutants and an isogenic W303 wild-type strain, grown either at 30 or 23°C, and analyzed the steady-state levels of pre-and mature rRNA species by northern blotting. As controls, we used the *asc1Δ* null mutant, which lacks the 40S r-protein Asc1 and the intron-encoded C/D-box snoRNA snR24 (66), and the *rps14A*[R136A] mutant, which displays a similar block in 20S pre-rRNA processing as the *rpl3*[W255C] mutant (67). As shown in Supplementary Figure S8A, a slight increase of the 35S pre-rRNA was detected in the *ssb1Δ ssb2Δ* mutant compared to the wild-type strain at both 30°C and 23°C (for a pre-rRNA processing scheme, see Supplementary Figure S1). This increase was accompanied by the appearance of the aberrant 23S pre-rRNA, which is more obvious at 23°C; however, no significant reduction of 20S pre-rRNAs was simultaneously observed. Analysis of low-molecular-weight RNA species showed that the levels of 7S pre-rRNAs and the mature 5S and 5.8S_S/L_ rRNAs remained practically unaffected (Supplementary Figure S8B). Altogether, these data indicate that Ssb1/2, and likely Zuo1 and Ssz1, play a modest role in early 35S pre-rRNA processing and, therefore, in 18S rRNA synthesis and 40S r-subunit biogenesis in the W303 background. Moreover, polysome profile analyses revealed that Ssb-RAC mutants display a modest but clear deficit in free 40S relative to 60S r-subunits, a hallmark of a 40S r-subunit shortage, which is consistent with defects in 18S rRNA synthesis (Supplementary Figure S9; see also (65)). In no case, the pre-rRNA processing phenotypes of the Ssb-RAC mutants were similar to those observed in the *rpl3*[W255C] (35) or the *rps14A*[R136A] mutant (67), which served as a positive control for 20S pre-rRNA accumulation.

**Figure 3.**
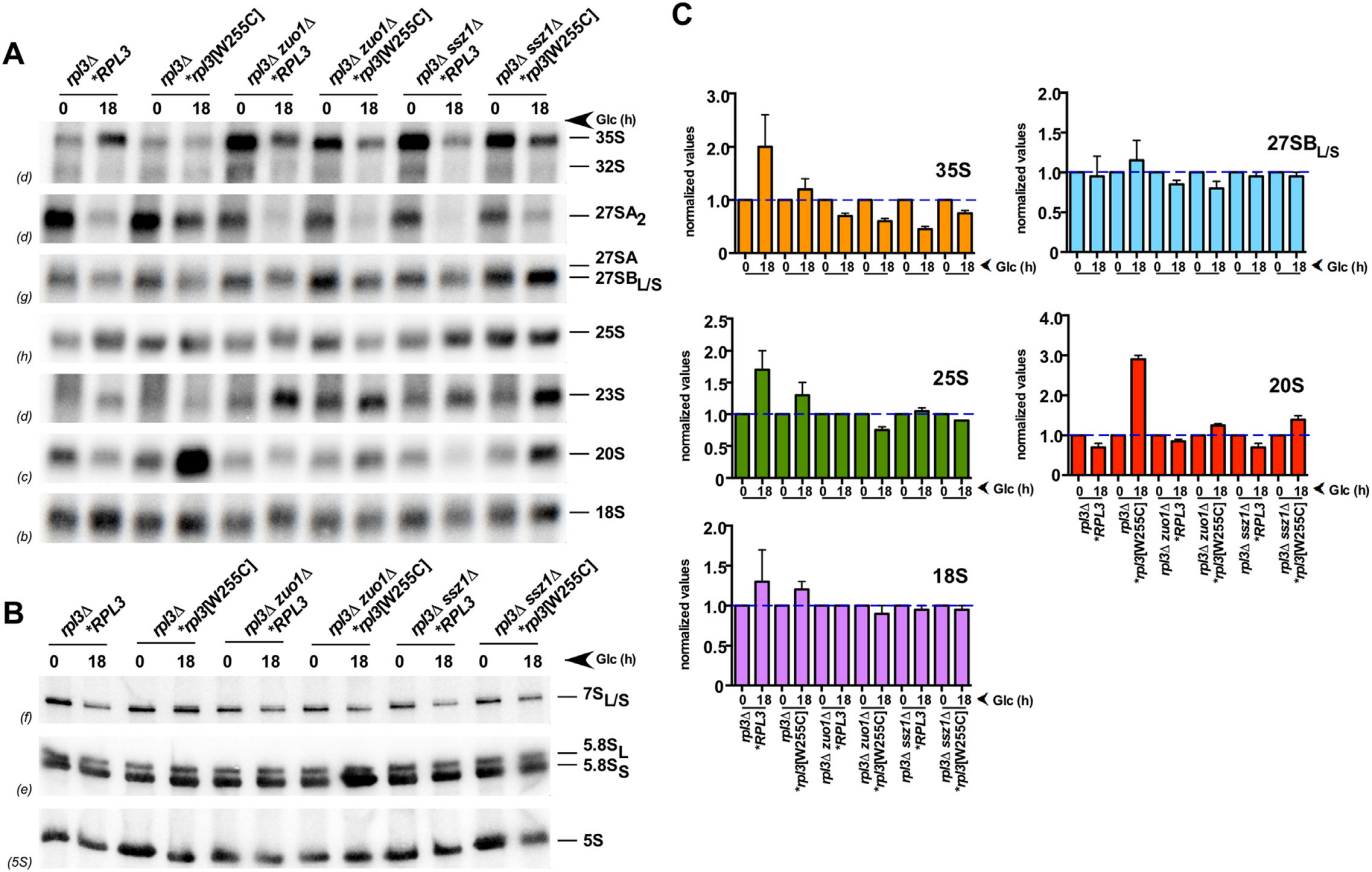
Pre-rRNA processing is not drastically perturbed in conditional *rpl3*[W255C] *zuo1Δ* and *rpl3*[W255C] *ssz1Δ* double mutants. The following strains were used: JDY1201 (rpl3Δ *RPL3), JDY1202 (rpl3Δ *rpl3[W255C]), JDY1203 (rpl3Δ zuo1Δ *RPL3), JDY1204 (rpl3Δ zuo1Δ *rpl3[W255C]), JDY1205 (rpl3Δ ssz1Δ *RPL3) and JDY1208 (rpl3Δ ssz1Δ *rpl3[W255C]). These strains harbor single *rpl3Δ* or double *rpl3Δ zuo1Δ* or *rpl3Δ ssz1Δ* genomic deletions, contain the *LEU2* plasmid pLCGTA-RPL3, which expresses wild-type *RPL3* under the transcriptional control of the *GAL1–10* promoter, and a *TRP1* plasmid harboring either wild-type *RPL3* or the mutant *rpl3*[W255C] allele. All strains were grown in SGal-Leu-Trp medium at 30°C to mid-exponential phase and then shifted to SD-Leu-Trp medium for 18 h at 30°C to deplete wild-type uL3 expressed from the *GAL1–10* promoter. Total RNA was extracted from each strain. Equal amounts of RNA (5 μg) were separated on (**A**) an 1.2% agarose–6% formaldehyde or on (**B**) *a* 7% polyacrylamide-8M urea gel, transferred to nylon membranes, and hybridized with the indicated probes (between parentheses; see Supplementary Table S3 for their location within the 35S pre-rRNA). (**C**) The signal intensities of each band were measured by phosphorimager scanning; values of pre- or mature rRNA species detected after the shift to SD-Leu-Trp medium were relativized to those obtained from samples grown in SGal-Leu-Trp medium, arbitrarily set at 1.0. The mean and standard deviations of three biological replicates (including the representative experiment presented in A) are shown.

To assess directly whether the synthetic lethality between the *rpl3*[W255C] allele and loss-of-function mutations in the Ssb-RAC chaperone triad is due to enhanced pre-rRNA processing defects, we constructed two conditional strains for phenotypic analyses. We first combined the *zuo1*Δ or the *ssz1*Δ deletions with a genomic *GAL::RPL3* allele; these strains express wild-type uL3 and grow as *zuo1*Δ or *ssz1*Δ strains, respectively, in SGal media but are unable to do so in SD media. Next, we transformed them with centromeric plasmids lacking an *RPL3* gene (empty plasmid) or either harboring wild-type *RPL3* or the *rpl3*[W255C] or *rpl3*[P257T] allele. As expected, the strains complemented with the *RPL3* or *rpl3*[P257T] alleles were able to grow on glucose-containing medium, while the growth of cells containing plasmid-borne *rpl3*[W255C] was severely compromised (Supplementary Figure S10). Then, we monitored pre-rRNA processing in the conditional *rpl3*[W255C] *zuo1Δ* and *rpl3*[W255C] *ssz1Δ* strains and their respective wild-type, single *rpl3*[W255C] and single *zuo1Δ* and *ssz1Δ* counterparts grown in selective SGal medium or after a shift to selective SD medium for 18 h at 30°C. After the shift, the growth rate of the conditional strains (*rpl3*[W255C] *zuo1Δ and rpl3*[W255C] *ssz1Δ*) rapidly decreased to a doubling time of >10 h after 18 h in glucose-containing media, while that of the respective control strains was roughly 1.5–2 h. As shown in Figure 3, pre-rRNA processing is not drastically perturbed in the double mutant strains, compared to the wild-type and single mutant controls. Rather than exhibiting an enhancement, the conditional strains notably showed a suppression of the prominent 20S pre-rRNA accumulation observed in the single *rpl3*[W255C] mutant (see Figure 3A and C). This finding was not unexpected, as we have previously reported that 20S pre-rRNA accumulation in *rpl3*[W255C] cells is dependent on active translation (35).

Taken together, we conclude that the lethality observed upon combination of the *rpl3*[W255C] allele with Ssb-RAC null mutations is not related to the role of this chaperone complex in ribosome biogenesis. In agreement, neither the *rps14A*[R136A], the *ltv1Δ* null, the *NOB1*-TAP nor the *ubi3Δub* allele, which all affect 20S pre-rRNA processing and cytoplasmic maturation of 40S r-subunits (35,67–69), significantly enhanced the sg phenotype of the *zuo1Δ* mutant (Supplementary Figures S11 and S12). As above, when pre-rRNA processing was studied, a suppression rather than an enhancement of the 20S pre-rRNA accumulation was observed for the double *ubi3Δub zuo1Δ* mutant compared to its single *ubi3Δub* counterpart (Supplementary Figure S13). Thus, absence of Ssb-RAC does not enhance the defects in ribosome biogenesis of strains with impaired cytoplasmic maturation of 20S pre-rRNA, including the *rpl3*[W255C] mutant.

### A defect in global protein folding cannot account for the genetic interaction between the *rpl3*[W255C] allele and loss-of-function mutations in Ssb-RAC

**Figure 4.**
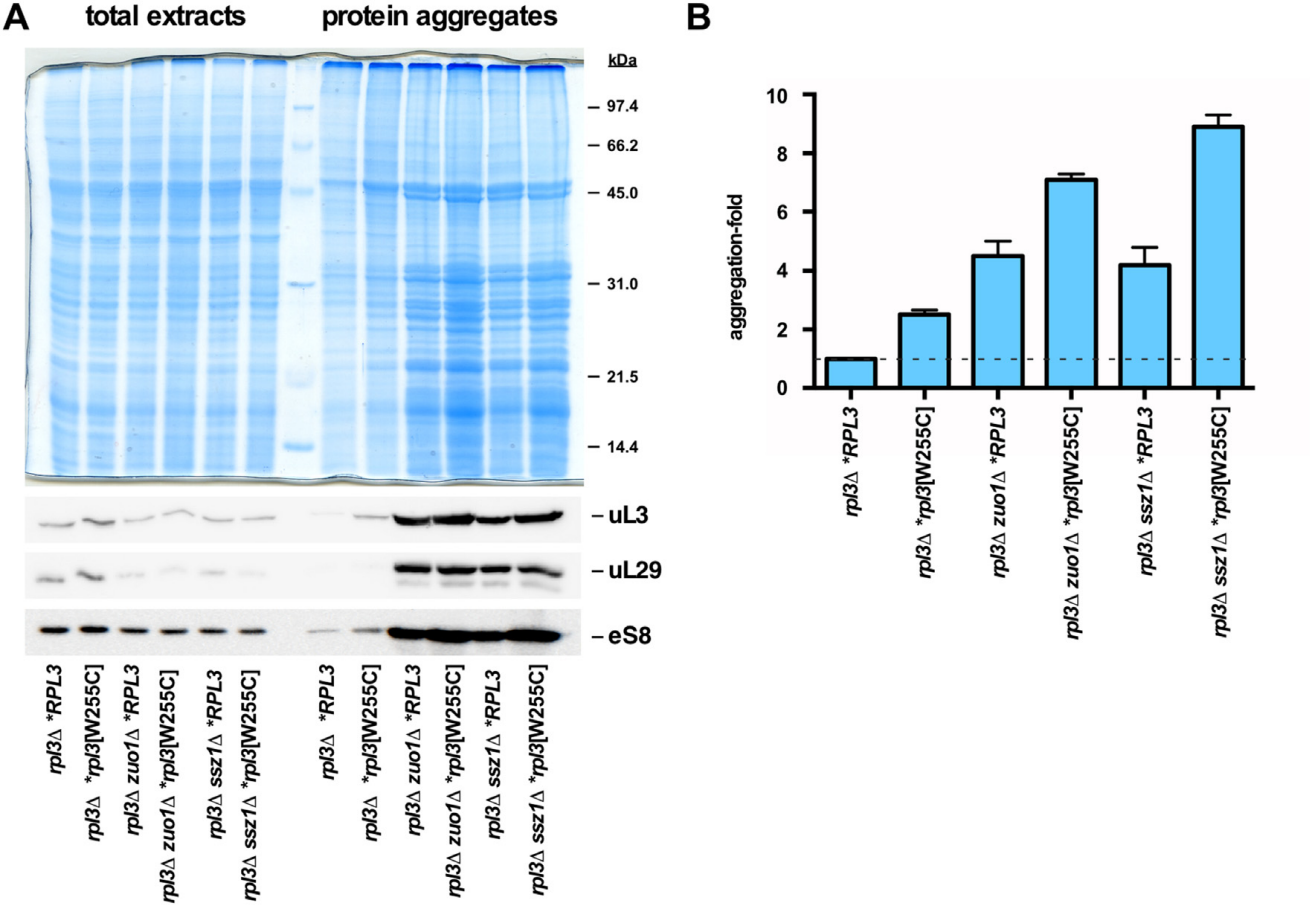
Protein aggregation in *rpl3*[W255C] cells in the presence or absence of Ssb-RAC. Total protein extracts and protein aggregates were prepared from the same strains described in the legend of Figure 3 and analyzed by SDS-PAGE and Coomassie staining or by western blotting with antibodies against uL3 uL29, and eS8. All strains were grown in SGal-Leu-Trp at 30°C to mid-exponential phase and then shifted to YPD for 18 h at 30°C to deplete wild-type uL3 expressed from the *GAL1–10* promoter. (**B**) Samples of protein aggregates were electrophoresed in 18-well Any kD™ Criterion™ TGX Stain-Free™ precast gels. Then, the lane profiles of the separated proteins loaded in each well were obtained using the Image Lab software provided for the ChemiDoc MP imaging system. The relative total protein intensity value for each profile was obtained and normalized to that of the wild-type situation (rpl3Δ *RPL3), which was arbitrarily set to 1.0. The mean and standard deviations of three biological replicates are shown.

Ribosome-associated chaperones prevent misfolding and protein aggregation. Indeed, it has been reported that cells lacking Ssb-RAC components exhibit extensive protein aggregation (22,24). Therefore, we examined whether the *rpl3*[W255C] mutation enhanced protein aggregation in a way that could explain the sl-relation between this allele and the Ssb-RAC mutants. To do so, we made again use of the conditional *rpl3*[W255C] *zuo1Δ* and *rpl3*[W255C] *ssz1Δ* strains and their respective wild-type, single *rpl3*[W255C], and single *zuo1Δ* and *ssz1Δ* counterparts. Cell extracts were prepared from cultures of these strains after a shift from selective SGal to YPD medium for 18 h at 30°C and the extent of protein aggregation was monitored. As expected (22), compared to the wild-type situation, similar levels of global protein aggregation were found in *zuo1Δ* and *ssz1Δ* cells (Figure 4). Protein aggregation was also found in *rpl3*[W255C] cells, a result that was similarly expected as defects in the ribosome biogenesis pathway lead to an increase in the amount of insoluble proteins (see (70,71)); however, the levels of global protein aggregation were slightly lower in *rpl3*[W255C] than in *zuo1Δ* or *ssz1Δ* cells (Figure 4). Importantly, the aggregation levels augmented in an additive or a mild synergistic manner in the conditional *rpl3*[W255C] *zuo1Δ* and *rpl3*[W255C] *ssz1Δ* strains, respectively (Figure 4B). However, we detected a practically identical pattern of protein aggregation in the different strains, suggesting that a similar subset of proteins was affected by the loss of Ssb-RAC components, regardless of the simultaneous presence of the *rpl3*[W255C] allele (see Figure 4A, gel). Moreover, we also found no apparent differences in the extent of aggregation of r-proteins, as exemplified by the detection of uL3, uL29, and eS8 by western blotting, in cells harboring either the *zuo1Δ* or the *ssz1Δ* mutation, independently of the uL3 source being either wild-type or uL3[W255C] variant protein (Figure 4A, blots). Altogether, these findings indicate that the lethality between *rpl3*[W255C] and Ssb-RAC null mutants can neither be attributed to an important enhancement of the protein aggregation propensity in the double mutants compared to the single mutants at the non-permissive condition nor to a particular increase of r-protein misfolding, which might alter the structural organization and function of ribosomes in the double mutant strains.

### The functional synergism between uL3 and Ssb-RAC is required for efficient translation

**Figure 5.**
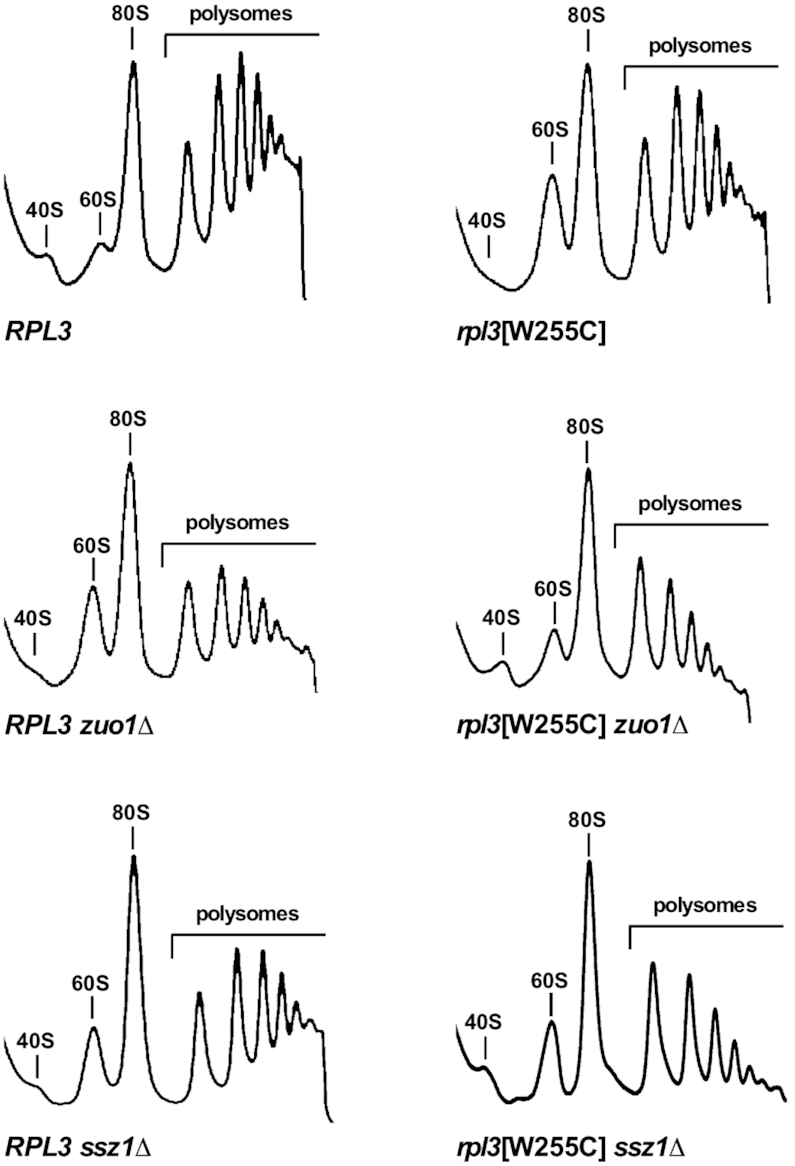
Translation elongation is impaired in *zuo1Δ* or *ssz1Δ* cells containing the *rpl3*[W255C] allele. The isogenic JDY1201 (*RPL3*), JDY1202 (*rpl3*[W255C]), JD1203 (*RPL3 zuo1Δ*), JDY1204 (*rpl3*[W255C] *zuo1Δ*), JDY1205 (*RPL3 ssz1Δ*) and JDY1208 (*rpl3*[W255C] *ssz1Δ*) strains, containing the *LEU2* plasmid pLCGTA-RPL3, which expresses wild-type *RPL3* under the transcriptional control of the *GAL1–10* promoter, and a *TRP1* plasmid harboring either wild-type *RPL3* or the *rpl3*[W255C] allele, were grown in selective SGal liquid medium at 30°C to mid-exponential phase. Then, the strains were shifted to selective SD liquid medium for 18 h at 30°C. Cells were harvested in mid-exponential growth phase, and cell extracts were prepared. Equivalent amounts of extracts (10 A_260_ units) were subjected to polysome profile analysis in 7–50% sucrose gradients. The *A*_254_ was continuously measured. Sedimentation is from left to right. The peaks of free 40S and 60S r-subunits, free 80S couples/monosomes, and polysomes are indicated.

As a third possibility, we explored whether the loss of Ssb-RAC synergistically enhanced the translational defects of the *rpl3*[W255C] mutant. To do so, we again made use of the conditional *rpl3*[W255C] *zuo1Δ* and *rpl3*[W255C] *ssz1Δ* strains. We first performed polysome profile analyses with cells extracts derived from the different strains after shifting them to selective SD for 18 h at 30°C. As shown in Figure 5, the strain expressing wild-type uL3 displayed a normal polysome profile when shifted to SD medium. Consistent with previous results (see Supplementary Figure S9 and (35)), the strains expressing uL3[W255C] or either lacking Zuo1 or Ssz1 exhibited profiles that are typical for mutants with a slight to moderate 40S r-subunit shortage (Figure 5). In comparison to these, the profiles obtained for the conditional *rpl3*[W255C] *zuo1Δ* and *rpl3*[W255C] *ssz1Δ* strains, which expressed uL3[W255C] as the sole uL3 source in either the background of a *zuo1Δ* or a *ssz1Δ* deletion, revealed an intriguing difference as we observed a successive decrease in the heights of the individual consecutive polysome peaks; thus, forming a descending slope from the di-some to the n-some peak, which is indicative of a lower ribosome density on the mRNA that may be due to a reduced rate of translation elongation. We therefore hypothesized that 80S initiation complexes containing uL3[W255C] may fail to optimally progress to the elongation phase of translation, especially when Ssb-RAC is simultaneously missing.

### The *rpl3*[W255C] mutation increases pausing of ribosomes early in translation elongation

**Figure 6.**
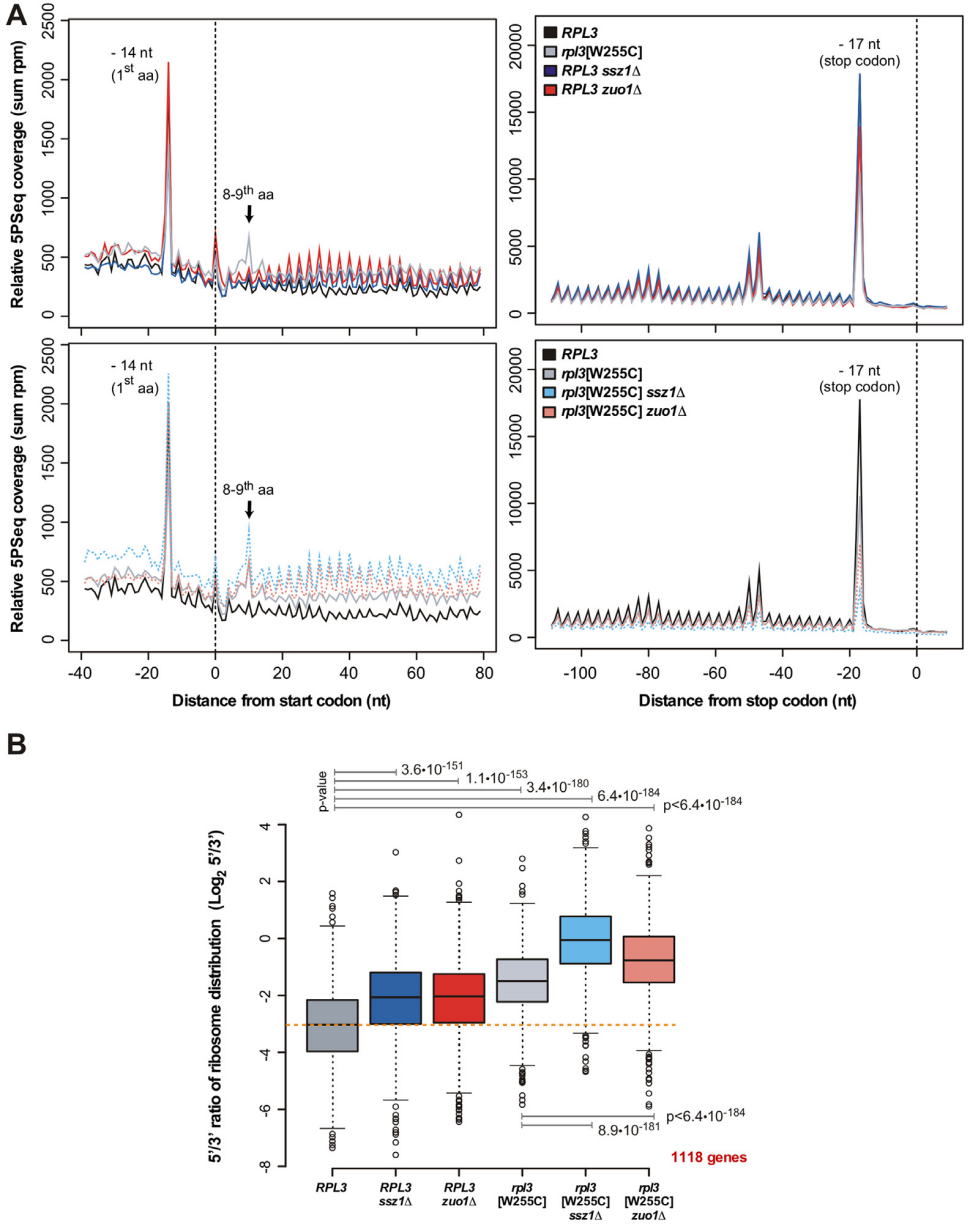
The translation elongation defect of *rpl3*[W255C] mutant cells is enhanced by the *zuo1Δ* or *ssz1Δ* mutations. (**A**) Metagene analysis displaying the abundance of 5′P intermediates in reads per million (sum rpm) around the start (left panels) and stop (right panels) codons. To decrease the effect of outliers, extreme values (top 5%) have been flattened down to 95%. (**B**) Relative 5PSeq coverage of the mRNA regions encoding the first *versus* the last 100 amino acids (log_2_ 5′/3′). Only genes whose mRNAs are long enough to encode 200 amino acids and covered by at least 10 5PSeq reads in each region have been considered (1118 genes in total). Significance of the mean ranks difference was assessed using a Wilcoxon paired signed-rank test. Note that as the 5PSeq analysis was performed with an oligo(dT) enrichment (72), a general increase in coverage towards the 3′ regions of the genes is expected. The used strains and growth conditions were as described in the legend of Figure 5.

To investigate whether *rpl3*[W255C] *zuo1Δ* and *rpl3*[W255C] *ssz1Δ* cells were indeed impaired in some aspect of translation elongation *in vivo*, we performed 5PSeq analysis. 5PSeq measures ribosome footprints at codon resolution by sequencing the intermediates of co-translational, Xrn1-dependent 5′-3′ mRNA degradation (72). We have previously established that ribosome occupancy generates an *in vivo* protection pattern with a clear 3-nucleotide (3-nt) periodicity, which allows to study translation at the level of initiation, elongation, and termination by focusing on mRNAs undergoing co-translational degradation (52,72). To this end, we prepared total RNA from the above *GAL::RPL3* strains, which were shifted for 18 h to selective SD medium before RNA extraction and 5PSeq analysis. In all cases, we could observe a clear 5PSeq peak at position –14-nt from the start codon, corresponding to ribosomes paused at the start codon with an initiator tRNA in the P-site (Figure 6A and Supplementary Figure S14). This result is consistent with the ribosome protection observed in a wild-type strain under specific growth conditions (i.e. cultures in stationary phase or treated with the translation elongation inhibitor cycloheximide) or in the *tif51A-3* mutant, which is impaired in translation termination (52,72). The ribosome protection within the 5′ regions of the transcripts was overall higher in the *rpl3*[W255C], *rpl3*[W255C] *zuo1Δ* and *rpl3*[W255C] *ssz1Δ* mutants than in the wild-type strain; this was evident when comparing for the same samples the ribosome protection pattern measured around the start and stop codon (Figure 6A). Consistently, this relative accumulation of ribosomes within the 5′ region of the mRNAs could also be confirmed by a relative decrease of ribosomal footprints associated with translation termination at the positions –17-nt and –47-nt from the stop codon, which are less prominent in the double *rpl3*[W255C] *zuo1Δ* and the *rpl3*[W255C] *ssz1Δ* conditional mutants than in the *rpl3*[W255C] mutant or the isogenic wild-type strain (Figure 6A and Supplementary Figure S14). Strikingly, in addition to the general increase in 5PSeq coverage within the 5′ regions of the open reading frames, we also identified an accumulation of 5PSeq reads 10-nt downstream of the start codon exclusively in all strains containing the *rpl3*[W255C] mutation (Figure 6A and Supplementary Figure S14). This peak corresponds to a ribosome paused at the ninth codon with a polypeptide-containing tRNA at the P-site, and thus, waiting for the accommodation of a charged aminoacyl-tRNA into the A-site at the 10th codon position. The biological relevance of this novel observation remains unclear; however, it must indicate a more frequent pausing or stalling of the translation elongation process at an early stage, which is likely due to an interaction of the short nascent peptides of nine amino acids with the PET of ribosomes specifically containing the uL3[W255C] r-protein. Interestingly, this phenomenon is increased when alanine or glycine are at the seventh or eighth position or tryptophan is at the ninth position of the nascent peptide (data not shown). Altogether, our data indicates that the *rpl3*[W255C] mutant is slightly impaired in translation elongation, a defect that is aggravated by the simultaneous absence of the RAC components Zuo1 or Ssz1.

To further support this conclusion, we computed the relative variation in the 5′ to 3′ ribosome protection pattern by comparing the ribosome protection associated with the region encoding the first and last 100 amino acids for each gene of sufficient length and sequence-read coverage (in total, 1118 genes) (see Figure 6B and its legend). The data confirmed that the single *rpl3*[W255C] mutant, and to a lesser extent the *zuo1Δ* and *ssz1Δ* mutants (see also, (23,73)), impair translation elongation (Figure 6B and Supplementary Figure S15). Importantly, this defect was even more evident when the *rpl3*[W255C] allele was combined with the *zuo1Δ* or the *ssz1Δ* null mutation (Figure 6B and Supplementary Figure S15). While this elongation defect can be observed for genes of all lengths, the relative ribosome accumulation within the 5′ coding region of the transcripts seems more pronounced for long genes, as would be expected for a translation elongation defect (Supplementary Figure S16).

Next, we addressed whether the 5PSeq features found for the conditional *rpl3*[W255C] *zuo1Δ* and *rpl3*[W255C] *szz1Δ* mutants were specific or shared by other mutations, such as *rps14A*[R136A], *ubi3Δub*, and *ltv1Δ*, that, as the *rpl3*[W255C] mutation, reduce 40S r-subunit production due to cytoplasmic accumulation of 20S pre-rRNA-containing pre-40S particles (67,69,74) (see also Supplementary Figure S13). Moreover, to make the comparison more significant, the *ubi3Δub* allele was also combined with the *zuo1Δ* null mutation. To do so, we employed the recently developed high-throughput HT-5PSeq protocol (54). As above, the single *zuo1Δ* mutant also showed with this procedure increased ribosome coverage within the proximal part of mRNAs compared to its isogenic wild-type counterpart (Supplementary Figure S17 and Figure 6). However, when a double *ubi3Δub zuo1Δ* mutant was assayed, a slight decrease in this coverage was found (Supplementary Figure S17). In addition, the HT-5PSeq profiles obtained for the *rps14A*[R136A] and *ltv1Δ* mutants were very similar to each other (Supplementary Figure S18). None of the above mutants showed an accumulation of 5PSeq reads 10-nt downstream of the start codon, which would indicate, as previously observed for *rpl3*[W255C] mutant cells, ribosome pausing at the ninth codon. Therefore, these results reinforce the fact that there is a specific translation elongation defect in *rpl3*[W255C] cells that is enhanced when Ssb-RAC is absent. In addition, uL3[W255C]-containing ribosomes make, in a Ssb-RAC independent manner, a distinct but discreet ribosome pausing at the ninth codon of mRNAs.

In conclusion, our data indicate that the Ssb-RAC complex is critical for the optimal functionality of ribosomes containing a specific PTC mutation; moreover, the interplay between uL3 and the Ssb-RAC chaperone triad during translation elongation likely contributes to the maintenance of cellular proteostasis.

## DISCUSSION

With this study, we have uncovered a hitherto unperceived functional link between translation elongation and nascent polypeptide folding by characterizing the negative impact elicited by a specific mutation in r-protein uL3 (*rpl3*[W255C] allele) in combination with the simultaneous absence of individual components of the ribosome-associated Ssb-RAC chaperone triad.

The conserved W255 residue of eukaryotic uL3 is of special interest since it represents the amino acid that is in closest proximity to the PTC and contributes to its correct structural organization, thus ensuring that the peptidyl-transfer reaction can proceed with the required efficiency (30). The *rpl3*[W255C] mutation, which causes an obvious widening of the tRNA-binding pocket at the A-site side of the PTC (30), leads to a range of phenotypes related to ribosome functionality (see Introduction), amongst them an increase in the affinity for aminoacyl- and peptidyl-tRNAs and a decrease in the affinity for eEF2 (75). In this work, we experimentally demonstrate, by using a genome-wide approach, that the *rpl3*[W255C] allele also causes a mild block of early translation elongation, as evidenced by an increased ribosome occupancy in the 5′ region of the translated open reading frames (Figure 6). The extent of this impairment is similar to the one observed for the single *zuo1Δ* and *ssz1Δ* mutants, but it is clearly much less pronounced than that reported by the same approach upon treatment with cycloheximide, which completely blocks translation elongation (72). Interestingly, it has been shown in human cells that the inhibition of Hsc70/Hsp70 (the functional homologue of Ssa proteins in mammals) also induces an early elongation pausing of ribosomes (26,27). In this case, it has been suggested that these chaperones fine-tune the translation elongation rate by co-translationally pulling the nascent protein chains out of the ribosomal exit tunnel. Thus, a decrease in the activity or availability of Hsc70/Hsp70 chaperones, either by their direct inactivation with small molecule inhibitors as VER-155008, by generation of proteotoxic stress with the proteasome inhibitor MG132 and/or AZC, or by a severe heat shock, is supposed to clog up the tunnel and reduce the elongation rate (27,73). Strikingly, our results unequivocally show that the combination of the specific *rpl3*[W255C] allele with the absence of the cytosolic Hsp70 chaperones Ssa1/2 or individual components of the ribosome-associated Ssb-RAC system (Ssb1/2, Zuo1 and Ssz1) causes a severe growth defect or lethality, respectively, which is, at least as shown to be the case for *rpl3*[W255C] mutant cells simultaneously lacking either Zuo1 or Ssz1, accompanied by an impairment of early translation elongation. However, it remains to be determined whether the observed elongation defect is the primary cause of the lethality of the double mutants (see below).

A central finding of this study is the allele specificity of the observed sl-interaction. Notably, there is no genetic interaction between the deletion of Ssa-class proteins or individual components of the Ssb-RAC complex and any of the other tested *rpl3* alleles. This is especially intriguing for the *rpl3*[P257T] and *rpl3*[I282T] alleles since they confer similar, albeit slightly less pronounced, defects in ribosome function (i.e. reduced translation, decreased peptidyl-transferase activity, anisomycin resistance, loss of killer phenotype, etc.) as the *rpl3*[W255C] mutation (56,76,77). Moreover, the conserved P257 residue of uL3 is located in the immediate proximity to W255; therefore, as already demonstrated for the *rpl3*[W255C] mutation (30), the *rpl3*[P257T] mutation may also be expected to alter the structure of the PTC. Nevertheless, we have previously also reported clear differences between these three alleles: the *rpl3*[W255C] mutation confers a net decrease in 40S r-subunit production and impairs processing of the 20S pre-rRNA into mature 18S rRNA, while the *rpl3*[P257T] mutation leads to a slight deficit of 60S r-subunits and the *rpl3*[I282T] mutation to a mild translation initiation defect (35). However, this remarkable observation is not sufficient to explain the sl-interaction with the *rpl3*[W255C] allele since our results indicate that W303-derived cells lacking either Zuo1, Ssz1 or Ssb1/2 affect 40S r-subunit biogenesis by only mildly impairing pre-rRNA processing at the early cleavage sites A_0_–A_2_ within the primary 35S pre-rRNA, but are not affected at all in 20S pre-rRNA processing. Accordingly, the combination between the *zuo1*Δ mutant and other mutant alleles (*NOB1*-TAP, *ltv1*Δ and *ubi3Δub*) that lead to defects in the cytoplasmic maturation of 18S rRNA does not result in a substantial enhancement of the respective growth defects (Supplementary Figures S11 and S12).

What is the reason for the severely impaired growth of *rpl3*[W255C] cells upon deletion of Ssb-RAC components or reduced activity of Ssa-class chaperones? Having discarded that the functional inactivation of the Ssb-RAC complex is synergistically enhancing the ribosome biogenesis defect of the *rpl3*[W255C] mutant (see Figure 3 and Supplementary Figure S8), we hypothesized that a translational shortcoming, especially affecting the translation of a subset of mRNAs encoding essential proteins whose sufficient abundance and functionality might be particularly dependent on a faithful translation or folding assistance, may be responsible for the fatal growth impairment. The types of translation defects observed for the *rpl3*[W255C] mutant are similar, yet slightly more pronounced, to those of the *rpl3*[P257T] or *rpl3*[I282T] mutants (see above); thus, it is possible that the critical ‘translational-deficiency’ threshold, below which mutants lacking Ssb-RAC or harboring reduced Ssa-chaperone activity can no longer grow, can theoretically only be reached by the *rpl3*[W255C] mutant. The affected translation-related processes might include: (i) Translational fidelity, as both the *rpl3* alleles and the deletion strains lacking Ssb-RAC components display decreased translational fidelity (73,78–81). (ii) Translation elongation, as shown in this study. It is possible that the levels of certain proteins, which are particularly dependent on the activity of the Ssb-RAC complex, are insufficient to support growth when translated by uL3[W255C]-containing ribosomes. (iii) Folding; it is known that the loss-of-function of Ssb-RAC components or deletion of *SSA1* results in protein aggregation (22,24), and that erroneous protein synthesis also promotes protein misfolding and aggregation (see (82), and references therein). Thus, it is conceivable that the combination of the *rpl3*[W225C] allele, which is prone to translational errors, with the deletion of Ssa1/2 or any component of the Ssb-RAC complex could aggravate the aggregation of particular newly synthesized proteins, which might result in cytotoxicity and cell death. It has also been proposed that the enhanced aggregation of nascent proteins, occurring in the absence of chaperones linked to protein synthesis (CLIPS), blocks the PET since the nascent proteins are not efficiently pulled out (73); thus, the nascent polypeptides move backwards and get clogged inside the tunnel, which in turn mispositions the peptidyl-tRNA in the P-site of the PTC and impedes the accommodation of the incoming aminoacyl-tRNA into the A-site (73). Accordingly, this model would fully explain why the lack of Ssb-RAC leads to an inhibition of the early phase of translation elongation (11,73). Given that global protein aggregation is not dramatically enhanced in the *rpl3*[W255C] *zuo1*Δ double mutant compared to cells lacking individual Ssb-RAC components, it might rather be the misfolding and aggregation of a specific subset of proteins that poses the deleterious problem. The uL3[W255C] protein itself is not part of this subset, as its mRNA shows a HT-5PSeq dynamics that is typical for this mRNA length (data not shown), and as its newly synthesized protein levels are similar in a wild-type or a *zuo1Δ* mutant strain (Supplementary Figure S5).

An alternative, non-mutually exclusive scenario that is worth being considered to explain the observed synthetic growth defect involves a direct and close physical coupling between the status of the PTC and the *de novo* folding of proteins at the solvent-side of the PET to ensure the production of correctly folded proteins. Under these circumstances, the loss of the coordination between these two strategical sites of the ribosome would interfere with protein homeostasis and therefore negatively affect cellular fitness and viability. Recent structural data are indeed supporting such a possibility as Zuo1 has been shown to interact both with the 60S r-subunit via helix 24 (H24) of the 25S rRNA, which is located at the exit site of the PET, and with the 40S r-subunit via expansion segment 12 (ES12) of the 18S rRNA (6,83,84). Strikingly, ES12 forms the solvent-facing tip of the long and functionally important helix 44 (H44), whose base is part of the decoding center (85). This dual binding mode is reminiscent of SRP, which interacts with the exit tunnel surface to scan for signal sequences and with the elongation factor binding site to slow down translation elongation (86,87). Moreover, a very recent study has reported an additional connection between the decoding center and the PTC of ribosomes and Ssb-RAC; thus, ribosomes assembled in the absence of Ssb-RAC appear to be structurally altered at these functional centers such that fidelity of translation termination is reduced (81). How exactly the Ssb-RAC complex, through the interaction of Zuo1 with both r-subunits, functionally monitors the progression of nascent chains inside the tunnel to coordinate translational activity with *de novo* protein folding and/or alters the structural features of ribosomes still awaits molecular clarification. This surveillance might start once nascent polypeptides are sufficiently long to be exposed and recognized by Ssb-RAC and/or Ssa (23,88).

In conclusion, current evidence implicates CLIPS as modulators of protein synthesis and cellular proteostasis. It has been shown by *in vivo* and *in vitro* experiments that misfolding of ribosome-bound nascent proteins attenuates early translation elongation and, *vice versa*, that the translation elongation rate affects protein folding ((23,27); reviewed in (11,28,89)). In these processes, the PET does not seem to act as a passive channel, instead, it forms a variable environment that interacts dynamically with the nascent polypeptides such that specific and intentional translational pausing can occur. Our study has revealed a novel, exciting example of a functional interaction between two distant ribosomal sites, the PTC and the distal end of the PET, and opens up the possibility to further explore this crosstalk between translation and folding in order to illuminate how CLIPS sense PTC activity.

## DATA AVAILABILITY

Raw and processed sequencing data were deposited at Gene Expression Omnibus (GEO) with accession number GSE114899 and GSE151632. Interactive browsable files can be accessed via the SciLifeLab data repository (https://figshare.com/s/9e695e6f6c1b97cd79ce).

## Supplementary Material

gkaa1200_Supplemental_File

## Notes

Present address: Juan J. García-Gómez, Cancer Research UK Drug-DNA Interactions Research Group, UCL Cancer Institute, University College London, London, UK.
