# Supplementary material for: A functional connection between translation elongation and protein folding at the ribosome exit tunnel in Saccharomyces cerevisiae
Source: Nucleic Acids Res. 2020 Dec 16;49(1):206–20. doi: 10.1093/nar/gkaa1200 (PMC7797049; doi:10.1093/nar/gkaa1200)
Supplement: gkaa1200_Supplemental_File [file gkaa1200_supplemental_file.docx]

**SUPPORTING INFORMATION**

**A functional connection between translation elongation and protein folding at the ribosome exit tunnel in *Saccharomyces cerevisiae***

Olga Rodríguez-Galán ^1,2,10^, Juan J. García-Gómez ^1,2,9,10^, Iván V. Rosado ^1,2^, Wu Wei ^3,4^, Alfonso Méndez-Godoy ^5^, Benjamin Pillet ^5^, Alisa Alekseenko ^6^, Lars M. Steinmetz ^3,7,8^, Vicent Pelechano ^6,11^, Dieter Kressler ^5,11^ and Jesús de la Cruz ^1,2,11^

^1^ Instituto de Biomedicina de Sevilla (IBiS), Hospital Universitario Virgen del Rocío/CSIC/Universidad de Sevilla, Seville, Spain. ^2^ Departamento de Genética, Universidad de Sevilla, Seville, Spain. ^3^ Stanford Genome Technology Center, Stanford University, Palo Alto, CA, USA. ^4^ CAS Key Lab of Computational Biology, CAS-MPG Partner Institute for Computational Biology, Shanghai Institute of Nutrition and Health, Shanghai Institutes for Biological Sciences, University of Chinese Academy of Sciences, Chinese Academy of Sciences, Shanghai, China. ^5^ Unit of Biochemistry, Department of Biology, University of Fribourg, Fribourg, Switzerland. ^6^ SciLifeLab, Department of Microbiology, Tumor and Cell Biology. Karolinska Institutet, Solna, Sweden. ^7^ European Molecular Biology Laboratory (EMBL), Genome Biology Unit, Heidelberg, Germany. ^8^ Department of Genetics, School of Medicine, Standord, CA, USA.

^9^ Current address: Cancer Research UK Drug-DNA Interactions Research Group, UCL Cancer Institute, University College London, London, UK.

**^10^ Co-first authors**

**^11^Co-corresponding authors**

**LEGENDS FOR SUPPLEMENTARY FIGURES**

**Figure S1.** Pre-rRNA processing pathway in *S. cerevisiae*. (***A***) Structure of an rDNA repeat unit. Each unit contains two independently transcribed elements, the long one is transcribed by RNA polymerase I (RNAP I) into a polycistronic pre-rRNA encoding the 18S, 5.8S, and 25S rRNAs and the short one is transcribed by RNA polymerase III (RNAP III) into a pre-5S rRNA. Non-transcribed, external, and internal spacers (NTSs, ETSs, and ITSs) are indicated. The mature rRNA species are shown as bars and the spacers as lines, thinner for NTSs. The transcription start sites are highlighted by red arrows and the processing sites indicated. Probes used for RNA hybridization, listed in Table S3, are also indicated. (***B***) Scheme of the pre-rRNA processing pathway. The RNAP I transcript can undergo either post- or co-transcriptional processing, leading to the generation of the 20S and 27SA_2_ pre-rRNAs, which are components of early pre-40S and pre-60S r-particles, respectively. These pre-rRNAs are then further processed into the mature 18S, 5.8S, and 25S rRNAs. The flanking 5S rRNA is transcribed by RNAP III in the opposite direction as a pre-5S rRNA. All cleavages and trimming reactions are indicated. This figure has been adapted from that shown in García-Gómez *et al.* (1). For further description of the yeast pre-rRNA processing pathway, see Fernández-Pevida *et al.* (2).

**Figure S2.** Genetic interaction between the *rpl3*[W255C] allele and mutations affecting different chaperone complexes involved in the *de novo* folding of proteins*.* (***A***) The *egd2∆* null mutation does not enhance the growth defect of the *rpl3*[W255C] mutant. A *rpl3∆* *egd2∆* double mutant (Y3593) and a reference *rpl3∆* strain (Y3594), both derived from the same diploid and containing wild-type *RPL3* on an *URA3* plasmid, were transformed with a *LEU2* plasmid harboring either wild-type *RPL3* or the indicated *rpl3* mutant alleles. After 5-FOA counter-selection, cells were spotted in 10-fold serial dilution steps onto YPD plates, which were incubated for 2 days at 30 ºC. (***B***) The *gim2∆* null mutation slightly enhances the growth defect associated with the *rpl3*[W255C] mutation. Growth of cells derived from a representative tetratype tetrad, resulting from a cross between a *gim2∆* (GMY11-8C) and a *rpl3*[W255C] (JDY318) mutant, is shown on a YPD plate, which was incubated for 3 days at 30 ºC. (***C***) The slow-growth phenotype of the *rpl3*[W255C] mutant is only very minorly enhanced by the *nat1∆* null mutation. A *rpl3∆* *nat1∆* double mutant (Y3605) and a reference *rpl3∆* strain (Y3604), both derived from the same diploid and containing wild-type *RPL3* on an *URA3* plasmid, were transformed with a *LEU2* plasmid harboring either wild-type *RPL3* or the mutant *rpl3*[Q371H] or *rpl3*[W255C] allele. After 5-FOA counter-selection, cells were spotted in 10-fold serial dilution steps onto YPD plates, which were incubated for 2 days at 30 ºC.

**Figure S3.** Specific synthetic lethal interaction between the *rpl3*[W255C] and the *ssa1∆* *ssa2∆* mutant. A *rpl3∆* *ssa1∆* *ssa2∆* triple mutant (ORY350), containing wild-type *RPL3* on an *URA3* plasmid, was transformed with a *LEU2* plasmid harboring either wild-type *RPL3* or the indicated *rpl3* mutant alleles. Cells were spotted in 5-fold serial dilution steps onto SD-Leu or SD+5-FOA plates, which were incubated for 4 days at 30 ºC.

**Figure S4.** The *rpl3*[W255C] mutant is extremely hypersensitive to azetidine-2-carboxylic acid (AZC), a drug that induces the misfolding of nascent proteins. Cells from isogenic wild-type (W303-1B), *rpl3*[W225C] (JDY318), *zuo1∆* (JDY509), and *ssz1∆* (JDY525) strains were spotted in 5-fold serial dilution steps onto YPD plates without (no drug) or supplemented with 1 mg/ml AZC (AZC), which were incubated for 3 days at 30 ºC.

**Figure S5**. Ribosomal protein uL3 is properly expressed in *zuo1∆* cells. Wild-type (W303-1B) or *zuo1∆* (YDK156-1C) cells were transformed with a plasmid harboring either the *RPL3*, *rpl3*[W255C] or *rpl3*[P257T] alleles under the control of the copper-inducible *CUP1* promoter. Cells were grown in SD-Leu medium lacking copper. Expression of the C-terminally 2xHA-tagged uL3, uL3[W255C] or uL3[P257T] proteins was induced for 20 min with 500 µM copper sulfate. After harvesting, total cell extracts were prepared, and equal amounts of protein were analyzed by SDS-PAGE and western blotting using anti-HA, anti-Adh1, and anti-Kar2 antibodies.

**Figure S6**. Ribosomal protein uL3[W255C] assembles into ribosomes, which engage in active translation. Wild-type (W303-1B) or *zuo1∆* (YDK156-1C) cells were transformed with a plasmid harboring either the *RPL3* or *rpl3*[W255C] alleles under the control of the copper-inducible *CUP1* promoter. Cells were grown in SD-Leu medium lacking copper. Expression of the C-terminally 2xHA-tagged uL3 or uL3[W255C] proteins was induced for 20 min with 500 μM copper sulfate. After harvesting, total cell extracts were prepared, and 10 A_260_ units of each extract were resolved in 7-50% sucrose gradients and fractionated. Proteins were extracted from each fraction and equal volumes analyzed by western blotting using anti-HA, anti-uL3, and anti-uL1 antibodies. Note that the anti-uL3 antibody reveals a doublet, which corresponds to the endogenous uL3 (lower band) and the induced uL3-2xHA (upper band, marked with an asterisk). The position of free 40S and 60S r-subunits, 80S vacant ribosomes/monosomes, and polysomes is indicated. T stands for total extract.

**Figure S7**. Consequences of the overexpression of the *rpl3*[W255C] allele. ***(A***) Increased dosage of *rpl3*[W255C] does not suppress its sl-relation with the *zuo1∆* allele. YDK157-2D cells (*zuo1∆ rpl3∆* [YCplac33-RPL3]), harboring a YCplac22-ZUO1 or an empty YCplac22 plasmid, were transformed with wild-type *RPL3* or the indicated *rpl3* mutant alleles, expressed from a low (YCplac111) or high (YEplac181) copy number plasmid. Cells were spotted in 10-fold serial dilution steps onto SD-Leu-Trp or SD-Leu-Trp+5-FOA plates, which were incubated for 4 days at 30 ºC. ***(B***) Increased dosage of *rpl3*[W255C] slightly enhances the growth defect of the *zuo1∆* mutant. YDK156-1A (wild type) and YDK156-1D (*zuo1∆*) cells were transformed with the indicated low- or high-copy plasmids. Cells were spotted in 10-fold serial dilution steps onto SD-Leu plates, which were incubated for 4 days at either 30 or 23 ºC.

**Figure S8.** Steady-state levels of pre-rRNAs and mature rRNAs in wild-type and *zuo1∆*, *ssz1∆*, *ssb1∆*, and *ssb1∆ ssb2∆* null mutant cells. Cells of the strains W303-1B, JDY509, JDY525, JDY534, and JDY532, respectively, were grown in YPD liquid medium at 30 or 23 ºC to mid-exponential phase. The *rps14A*[R136A] (JDY546) and *asc1∆* (JDY539) mutants were used as controls. Total RNA was extracted from each strain. Equal amounts of RNA (5 µg) were separated on (***A***) an 1.2% agarose-6% formaldehyde or on (***B***) *a* 7% polyacrylamide-8M urea gel, transferred to nylon membranes, and hybridized with the indicated probes (between parentheses; see Table S3 for their location within the 35S pre-rRNA). Note that the *rps14A*[R136A] mutant, similarly to the *rpl3*[W255C] mutant, accumulates 20S pre-rRNA (1,3).

**Figure S9.** Deletion of *ZUO1*, *SSZ1* or *SSB1/2* results in a slight deficit in 40S r-subunits. Polysome profiles are shown for isogenic wild-type (W303-1B), *zuo1∆* (JDY509), *ssz1∆* (JDY525)*,* and *ssb1∆ ssb2∆* (JDY532) cells grown in YPD medium at 30 ºC to mid-exponential phase. Cells were harvested and extracts were prepared. Ten A_260_ units of each extract were resolved in 7-50% sucrose gradients and the A_254_ was continuously measured. Sedimentation is from left to right. The peaks of free 40S and 60S r-subunits, free 80S couples/monosomes, and polysomes are indicated.

**Figure S10.** Conditional systems for the phenotypic analysis of *rpl3*[W255C] *zuo1∆* and *rpl3*[W255C] *ssz1∆* double mutants. Double *rpl3∆* *zuo1∆* (JDY1203) and *rpl3∆* *ssz1∆* (JDY1205) mutants containing the *LEU2* plasmid pLCGTA-RPL3, which expresses wild-type *RPL3* under the transcriptional control of the *GAL1-10* promoter, were transformed with *TRP1* plasmids harboring either wild-type *RPL3* or mutant *rpl3*[W255C] or *rpl3*[P257T] alleles. The cells were grown on SGal-Leu-Trp and then streaked on a SD-Leu-Trp plate, which was incubated at 30 ºC for 5 days.

**Figure S11.** Mutations affecting cytoplasmic 20S pre-rRNA processing do not confer synthetic lethality to *zuo1∆* null mutant cells. (***A***) A *rps14A∆* *rps14B∆* *zuo1∆* triple mutant (Y4504) and a reference *rps14A∆* *rps14B∆* strain (Y4503), both derived from the same diploid and containing wild-type *RPS14A* on an *URA3* plasmid, were transformed with an empty *LEU2* plasmid or the same plasmid harboring either wild-type *RPS14A* or the *rps14A*[R136A] mutant allele. Cells were spotted in 10-fold serial dilution steps onto SD-Leu or SD+5-FOA plates, which were incubated for 3 or 4 days at 30 ºC, respectively. (***B***) Growth of cells derived from a representative tetratype tetrad, resulting from a cross between a *NOB1*-TAP strain (YKL199) and a *zuo1∆* mutant (YDK156-1C), is shown on a YPD plate, which was incubated for 2 days at 30 ºC. (***C***) Growth of cells derived from a representative tetratype tetrad, resulting from a cross between a *ltv1∆* (YKL1356) and a *zuo1∆* (YDK156-1C) mutant, is shown on a YPD plate, which was incubated for 2 days at 30 ºC.

**Figure S12**. The *ubi3∆ub∆* null mutation does not significantly enhance the growth defect of the *zuo1∆* mutant. Growth of cells derived from a representative tetratype tetrad, resulting from a cross between a *ubi3∆ub* (TLY61.A2) and a *zuo1∆* (JDY509) mutant, is shown on a YPD plate, which was incubated for 3 days at 30 ºC.

**Figure S13**. Steady-state levels of pre-rRNAs and mature rRNAs in wild-type and *zuo1∆*, *ubi3∆ub*, *ubi3∆ub zuo1∆*, *ltv1∆*, and *rps14A*[R136A] mutant cells. Cells of the strains W303-1B, JDY509, TLY61.A2, JDY1180, YKL1356 and JDY544, respectively, were grown in SD liquid medium at 30 ºC to mid-exponential phase. Total RNA was extracted from each strain. Equal amounts of RNA (5 µg) were separated on (***A***) an 1.2% agarose-6% formaldehyde or on (***B***) *a* 7% polyacrylamide-8M urea gel, transferred to nylon membranes, and hybridized with the indicated probes (between parentheses; see Table S3 for their location within the 35S pre-rRNA). Note that the *ubi3∆ub*, *ubi3∆ub zuo1∆,* *ltv1∆* and *rps14A*[R136A] mutants, similarly to the *rpl3*[W255C] mutant, accumulates 20S pre-rRNA ((3-5); this work).

**Figure S14.** Detailed metagene analysis displaying the relative coverage of 5PSeq reads per intermediate (in reads per million; sum rpm) with respect to start (left panels) and stop (right panels) codons. Note that for visual convenience, we compared in the different panels wild-type *versus* *rpl3*[W255C], single *ssz1∆* *versus* double *rpl3*[W255C] *ssz1∆*, and single *zuo1∆* *versus* double *rpl3*[W255C] *zuo1∆* cells. See Figure 6 for further details.

**Figure S15.** The *rpl3*[W255C] mutation leads to the accumulation of ribosome protection in the 5' region of mRNAs, and combination with the *zuo1∆* or the *ssz1∆* mutation enhances this effect. (***A***) Density distribution of genes with respect to the relative 5PSeq coverage of the mRNA region encoding the first *versus* the last 100 amino acids (Log_2_ 5'/3'). Note that strains containing *rpl3*[W255C], *ssz1∆,* or *zuo1∆* alleles and their combinations present an increased 5PSeq coverage in the 5’ region of the genes (higher Log_2_ 5'/3'). As in Figure 6B, only genes whose mRNAs are long enough to encode 200 amino acids and covered by at least ten 5PSeq reads in each region have been considered (1118 genes in total). (***B***) Detailed view of the accumulation of ribosome protection in the 5' region analyzed at gene-specific level. Specifically, the Log_2_ 5'/3' ratio for 5PSeq coverage shown at population level in ***A***, is shown as a scatter plot (one point per analyzed gene). The correlation across samples suggest that an accumulation of ribosomes within the 5´regions is a general effect affecting the bulk of the transcriptome.

**Figure S16.** The 5' accumulation of ribosomes in the conditional *rpl3*[W255C] *ssz1∆* and *rpl3*[W255C] *zuo1∆* double mutant strains affects mRNAs of all gene lengths. Relative 5PSeq coverage of the mRNA region encoding the first *versus* the last 100 amino acids (Log_2_ 5'/3'). Genes are those considered in Figure 6B, which were ranked as short (S), mid-short (MS), mid-long (ML), and long (L) according to their length. See Figure 6 for further details.

**Figure S17.** Detailed metagene analysis displaying the relative coverage of HT-5PSeq reads per intermediate (in reads per million; sum rpm) with respect to start (left panels) and stop (right panels) codons. Note that for visual convenience, we compared in the different panels wild-type *versus* single *zuo1∆,* single *ubi3∆ub* *versus* double *ubi3∆ub* *zuo1∆* cells*,* and simultaneously cells of the corresponding four strains. See Figure 6 for further details.

**Figure S18.** Detailed metagene analysis displaying the relative coverage of HT-5PSeq reads per intermediate (in reads per million; sum rpm) with respect to start (left panels) and stop (right panels) codons. Note that for visual convenience, we compared in the different panels wild-type *versus* *ltv1∆,* wild-type *versus* *rpl14A*[R136A] cells, and simultaneously cells of the corresponding three strains. See Figure 6 for further details.

**Table S1. Yeast strains used in this study.**

| **Strain** | **Relevant genotype** | **Source** |
| --- | --- | --- |
| W303-1A | *MAT***a** *ade2-1 his3-11,15 leu2-3,112 trp1-1 ura3-1* | (6) |
| W303-1B | As W303-1A but *MAT*α | (6) |
| YDK39-1D ^(a)^ | *MAT***a** *rpl3*::kanMX4 [YCplac33-RPL3] | This study |
| YDK39-1C ^(a)^ | *MAT*α *rpl3*::kanMX4 [YCplac33-RPL3] | This study |
| JDY318 ^(a)^ | *MAT*α *rpl3*::HIS3MX6 [YCplac33-RPL3] | (7) |
| YDK145-7D ^(a)^ | *MAT***a** *rpl3*::kanMX4 *ade3*::kanMX4 [pHT4467∆-RPL3] [YCplac22-rpl3-W255C] | This study |
| JDY457 | *MAT***a** *rpl3*::kanMX4 *ade3*::kanMX4 [pHT4467∆-RPL3] [YCplac22-rpl3-W255C] *sl7D30/2* | This study |
| JDY509 | *MAT***a** *zuo1*::HIS3MX6 | This study |
| YDK156-1A | As W303-1A | This study |
| YDK156-1C | *MAT*α *zuo1*::HIS3MX6 | This study |
| YDK156-1D | *MAT***a** *zuo1*::HIS3MX6 | This study |
| YDK157-2B ^(a)^ | *MAT***a** *rpl3*::kanMX4 [YCplac33-RPL3] | This study |
| YDK157-2D ^(a)^ | *MAT***a** *zuo1*::HIS3MX6 *rpl3*::kanMX4 [YCplac33-RPL3] | This study |
| JDY525 | *MAT***a** *ssz1*::HIS3MX6 | This study |
| YKD9-6C ^(a)^ | *MAT*α *ssz1*::HIS3MX6 *rpl3*::kanMX4 [YCplac33-RPL3] | This study |
| JDY534 | *MAT***a** *ssb1*::HIS3MX6 | This study |
| JDY532 | *MAT***a** *ssb1*::HIS3MX6 *ssb2*::natNT2 | This study |
| YKD7-9D ^(a)^ | *MAT*α *ssb1*::HIS3MX6 *ssb2*::natNT2 *rpl3*::kanMX4 [YCplac33-RPL3] | This study |
| YKD7-9A ^(a)^ | *MAT***a** *ssb2*::natNT2 *rpl3*::kanMX4 [YCplac33-RPL3] | This study |
| JDY546 | *MAT***a** *rps14A*::HIS3MX6 rps14B::natNT2 [YCplac22-rps14A-R136A] | (1) |
| JDY539 | *MAT*α *asc1::* HIS3MX6 | This study |
| Y3593 ^(a)^ | *MAT***a** *egd2*::HIS3MX6 *rpl3*::kanMX4 [YCplac33-RPL3] | This study |
| GMY11-8C | *MAT***a** *gim2*::kanMX4 | This study |
| Y3605 ^(a)^ | *MAT***a** *nat1*::HIS3MX6 *rpl3*::kanMX4 [YCplac33-RPL3] | This study |
| TR125 | *MAT***a** *ssa1::HIS3 ssa2::LEU2* | (8) |
| ORY350^(a)^ | *MAT***a** *ssa1::HIS3 ssa2::LEU2 rpl3*::kanMX4 [YCplac33-RPL3] | This study |
| JDY544 ^(b)^ | *MAT***a** *rps14A*::HISMX6 rps14B::natNT2 [YCplac33-RPS14A] | This study |
| Y4504 ^(b)^ | *MAT***a** *zuo1*::HIS3MX6 *rps14A*::HISMX6 rps14B::natNT2 [YCplac33-RPS14A] | This study |
| YKL199 | *MAT***a** *NOB1*-TAP::natNT2 | This study |
| YKL1356 | *MAT***a** *ltv1*::klTRP1 | This study |
| TLY61.A2 | *MAT*α *ubi3∆ub*-HA::kanMX6 | (4) |
| JDY1180 | *MAT***a** *zuo1*::HIS3MX6 *ubi3∆ub*-HA::kanMX6 | This study |
| JDY1201 | *MAT*α *rpl3*::kanMX4 [pLCGTA-RPL3] [YCplac22-RPL3] | This study |
| JDY1202 | *MAT*α *rpl3*::kanMX4 [pLCGTA-RPL3] [YCplac22-rpl3-W255C] | This study |
| JDY1203 | *MAT*α *zuo1*::HIS3MX6 *rpl3*::kanMX4 [pLCGTA-RPL3] [YCplac22-RPL3] | This study |
| JDY1204 | *MAT*α *zuo1*::HIS3MX6 *rpl3*::kanMX4 [pLCGTA-RPL3] [YCplac22-rpl3-W255C] | This study |
| JDY1205 | *MAT*α *ssz1*::HIS3MX6 *rpl3*::kanMX4 [pLCGTA-RPL3] [YCplac22-RPL3] | This study |
| JDY1208 | *MAT*α *ssz1*::HIS3MX6 *rpl3*::kanMX4 [pLCGTA-RPL3] [YCplac22-rpl3-W255C] | This study |

^(a)^ These strains require a plasmid-borne *RPL3* allele to support growth. Depending on the experimental conditions and as indicated in the text, this plasmid might be different to YCplac33-RPL3 or pHT4467∆-RPL3.

^(b)^ These strains require a plasmid-borne *RPS14* allele to support growth. Depending on the experimental conditions and as indicated in the text, this plasmid might be different to YCplac33-RPS14A.

**Table S2. Plasmids used in this study.**

| **Name** | **Relevant information** | **Source** |
| --- | --- | --- |
| YCplac33 | *CEN*, *URA3* | (9) |
| YCplac33-RPL3 | *RPL3*, *CEN*, *URA3* | (7) |
| YCplac33-RPS14A | *RPS14A*, *CEN*, *URA3* | This study |
| YCplac22 | *CEN*, *TRP1* | (9) |
| YCplac22-RPL3 | *RPL3*, *CEN*, *TRP1* | (7) |
| YCplac22-rpl3-Q371H | *rpl3*[Q371H], *CEN*, *TRP1* | (7) |
| YCplac22-rpl3-K30E | *rpl3*[K30E], *CEN*, *TRP1* | (7) |
| YCplac22-rpl3-W255C | *rpl3*[W255C], *CEN*, *TRP1* | (7) |
| YCplac22-rps14A-R136A | *rps14A*[R136A], *CEN*, *TRP1* | (1) |
| YCplac111 | *CEN*, *LEU2* | (9) |
| YCplac111-RPL3 | *RPL3*, *CEN*, *LEU2* | (7) |
| YCplac111-rpl3-W255C | *rpl3*[W255C], *CEN*, *LEU2* | (1) |
| YCplac111-rpl3-Q371H (YCplac111-rpl3-101) | *rpl3*[Q371H], *CEN*, *LEU2* | (1) |
| YCplac111-rpl3-K30E (YCplac111-rpl3-102) | *rpl3*[K30E], *CEN*, *LEU2* | (1) |
| YCplac111-rpl3-P257T | *rpl3*[P257T], *CEN*, *LEU2* | (1) |
| YCplac111-rpl3-I282T | *rpl3*[I282T], *CEN*, *LEU2* | (1) |
| YCplac111-RPS14A | *RPS14A*, *CEN*, *LEU2* | This study |
| YCplac111-rps14A-R136A | *rps14A*[R136A], *CEN, LEU2* | This study |
| YCplac111-ZUO1 | *ZUO1*, *CEN*, *LEU2* | This study |
| YEplac181 | *2µ*, *LEU2* | (9) |
| YEplac181-ZUO1 | *ZUO1*, *2µ*, *LEU2* | This study |
| YEplac181-RPL3 | *RPL3, 2µ*, *LEU2* | This study |
| YEplac181-rpl3-W255C | *rpl3*[W255C], *2µ*, *LEU2* | This study |
| YEplac181-rpl3-P257T | *rpl3*[P257T], *2µ*, *LEU2* | This study |
| pHT4467∆-RPL3 | *RPL3*, *CEN6 (instable*), *URA3*, *ADE3* | (1) |
| pLCGTA-RPL3 | P*GAL*, *RPL3*, T*ADH1*, *CEN*, *LEU2* | This study |
| pCUP111-RPL3-2HA | P*CUP1*, *RPL3-*2xHA, T*ADH1*, *CEN*, *LEU2* | This study |
| pCUP111-rpl3-W255C-2HA | P*CUP1*, *rpl3*[W255C]-2xHA, T*ADH1*, *CEN*, *LEU2* | This study |
| pCUP111-rpl3-P257T-2HA | P*CUP1*, *rpl3*[P257T]-2xHA, T*ADH1*, *CEN*, *LEU2* | This study |

**Table S3. Oligonucleotides used in this study.**

| **Name** | **5'-3' Sequence** | **Use** |
| --- | --- | --- |
| Probe a (5' A_0_) | GGTCTCTCTGCTGCCGG | Pre-rRNA hybridization |
| Probe b (18S) | CATGGCTTAATCTTTGAGAC | 18S rRNA hybridization |
| Probe c (3-D/A_2_) | GACTCTCCATCTCTTGTCTTCTTG | Pre-rRNA hybridization |
| Probe d (A_2_/A_3_) | TGTTACCTCTGGGCCC | Pre-rRNA hybridization |
| Probe e (5.8S) | TTTCGCTGCGTTCTTCATC | 5.8S rRNA hybridization |
| Probe f (E/C_2_) | GGCCAGCAATTTCAAGTTA | Pre-rRNA hybridization |
| Probe g (C_1_/C_2_) | GAACATTGTTCGCCTAGA | Pre-rRNA hybridization |
| Probe h (25S) | CTCCGCTTATTGATATGC | 25S rRNA hybridization |
| Probe 5S | GGTCACCCACTACACTACTCGG | 5S rRNA hybridization |

**SUPPLEMENTARY REFERENCES**

1. García-Gómez, J.J., Fernández-Pevida, A., Lebaron, S., Rosado, I.V., Tollervey, D., Kressler, D. and de la Cruz, J. (2014) Final pre-40S maturation depends on the functional integrity of the 60S subunit ribosomal protein L3. *PLoS Genet.*, **10**, e1004205.

2. Fernández-Pevida, A., Kressler, D. and de la Cruz, J. (2015) Processing of preribosomal RNA in *Saccharomyces cerevisiae*. *Wiley Interdiscip. Rev. RNA*, **6**, 191-209.

3. Jakovljevic, J., de Mayolo, P.A., Miles, T.D., Nguyen, T.M., Léger-Silvestre, I., Gas, N. and Woolford, J.L., Jr. (2004) The carboxy-terminal extension of yeast ribosomal protein S14 is necessary for maturation of 43S preribosomes. *Mol. Cell*, **14**, 331-342.

4. Lacombe, T., García-Gómez, J.J., de la Cruz, J., Roser, D., Hurt, E., Linder, P. and Kressler, D. (2009) Linear ubiquitin fusion to Rps31 and its subsequent cleavage are required for the efficient production and functional integrity of 40S ribosomal subunits. *Mol. Microbiol.*, **72**, 69-84.

5. Seiser, R.M., Sundberg, A.E., Wollam, B.J., Zobel-Thropp, P., Baldwin, K., Spector, M.D. and Lycan, D.E. (2006) Ltv1 is required for efficient nuclear export of the ribosomal small subunit in *Saccharomyces cerevisiae*. *Genetics*, **174**, 679-691.

6. Thomas, B.J. and Rothstein, R. (1989) Elevated recombination rates in transcriptionally active DNA. *Cell*, **56**, 619-630.

7. de la Cruz, J., Lacombe, T., Deloche, O., Linder, P. and Kressler, D. (2004) The putative RNA helicase Dbp6p functionally interacts with Rpl3p, Nop8p and the novel trans-acting factor Rsa3p during biogenesis of 60S ribosomal subunits in *Saccharomyces cerevisiae*. *Genetics*, **166**, 1687-1699.

8. Xi, W., Wang, X., Laue, T.M. and Denis, C.L. (2016) Multiple discrete soluble aggregates influence polyglutamine toxicity in a Huntington's disease model system. *Sci. Rep.*, **6**, 34916.

9. Gietz, R.D. and Sugino, A. (1988) New yeast-*Escherichia coli* shuttle vectors constructed with *in vitro* mutagenized yeast genes lacking six-base pair restriction sites. *Gene*, **74**, 527-534.


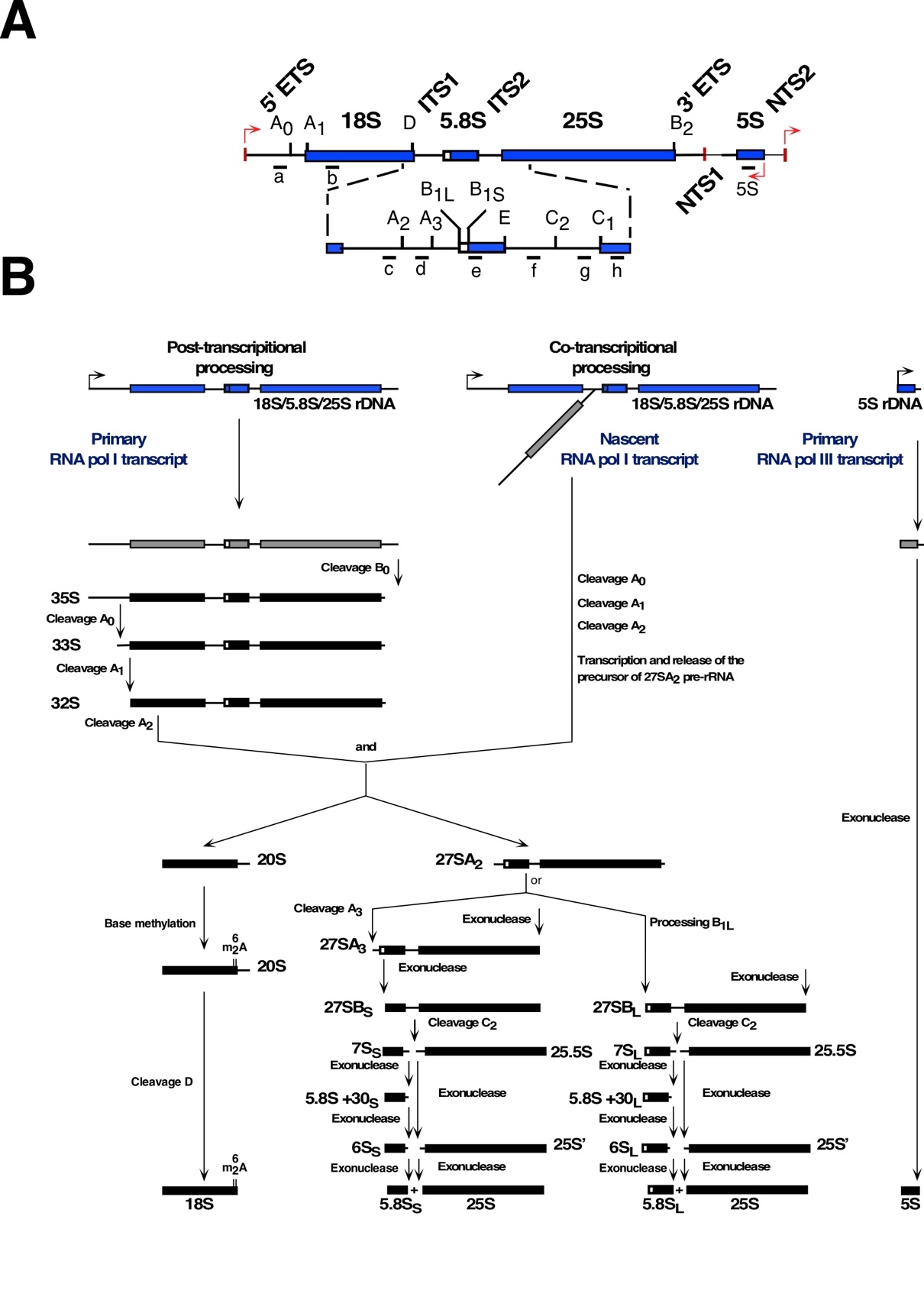


**FIGURE S1. Rodríguez-Galán *et al.***


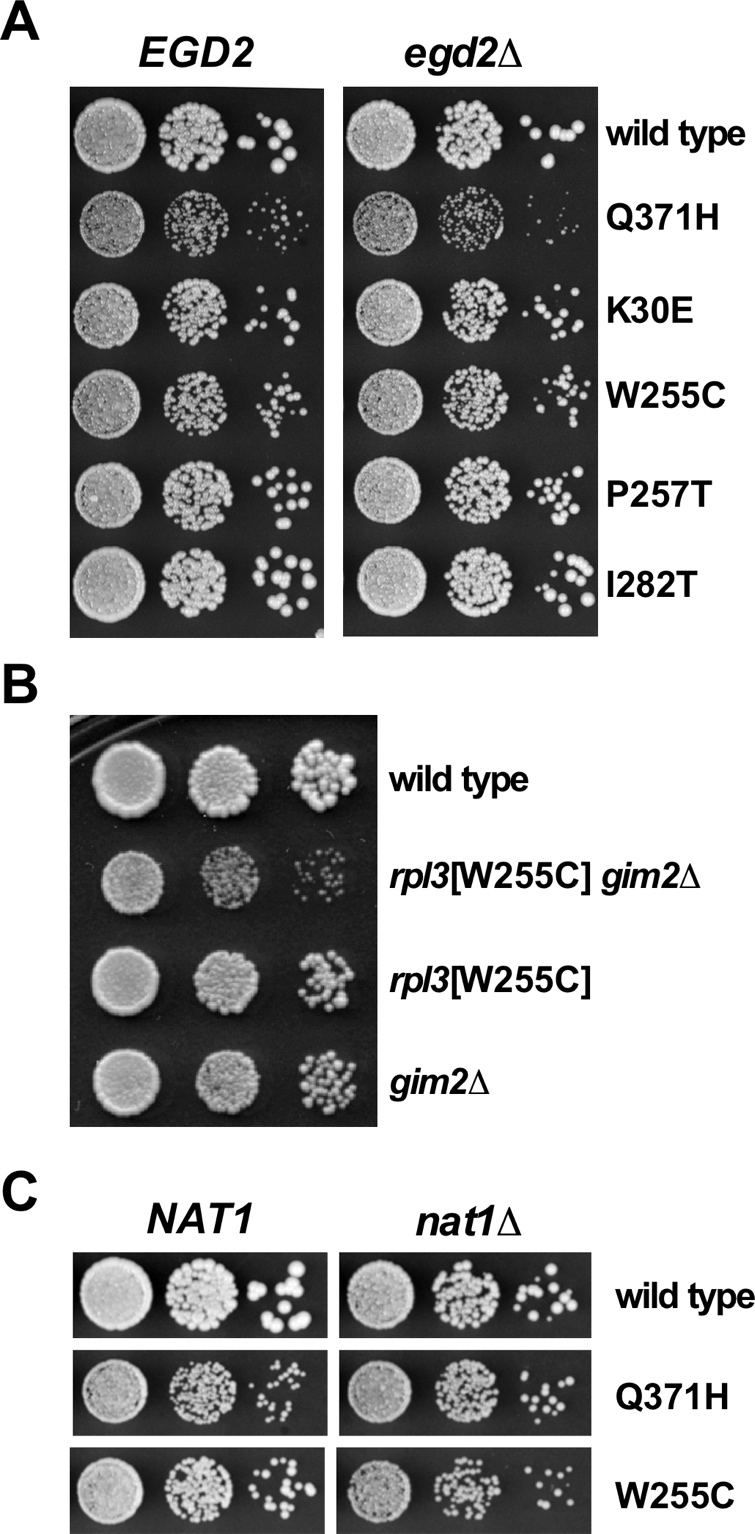


**FIGURE S2. Rodríguez-Galán *et al.***

**
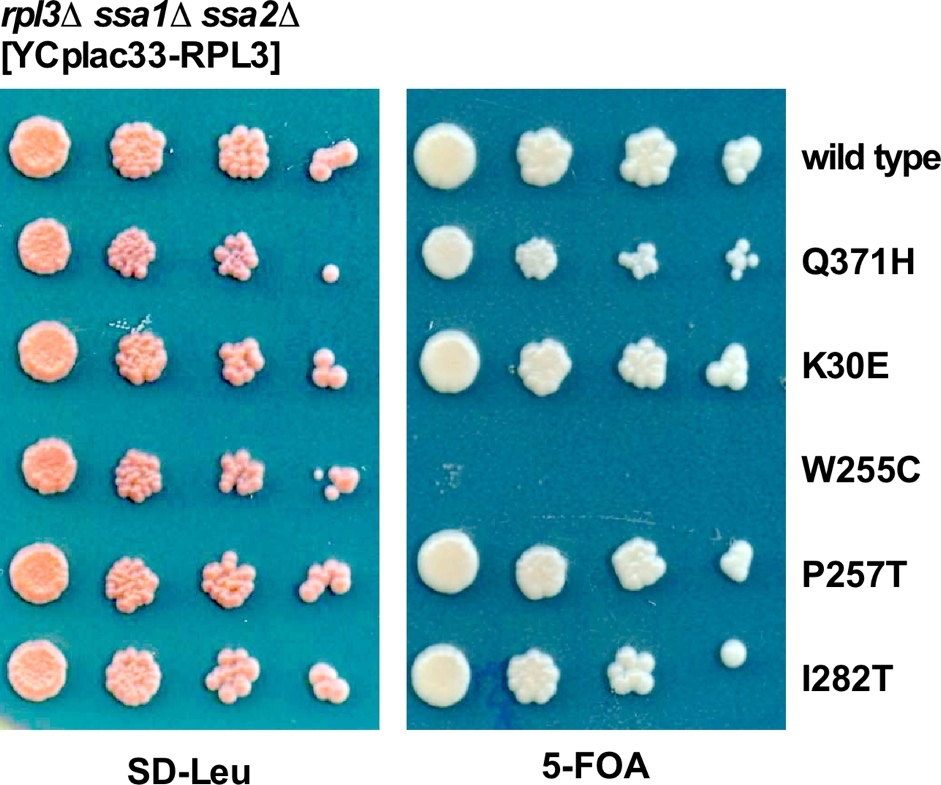
**

**FIGURE S3. Rodríguez-Galán *et al.***


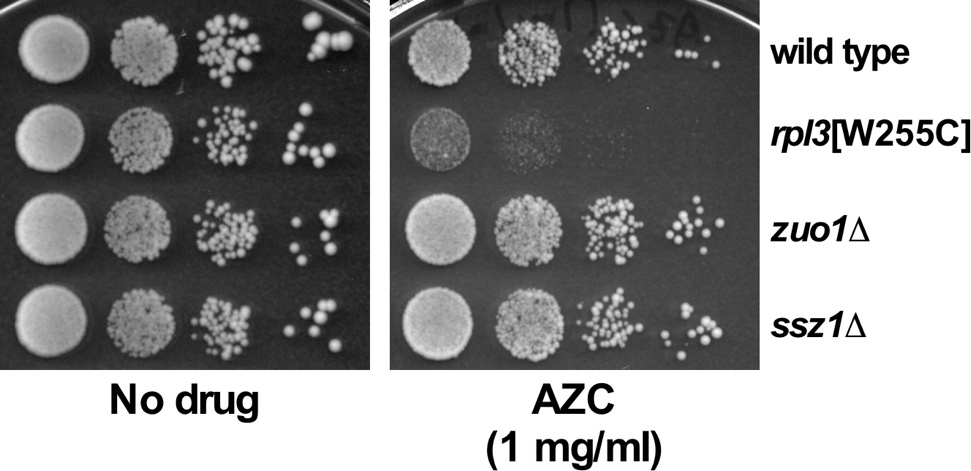


**FIGURE S4. Rodríguez-Galán *et al.***

**
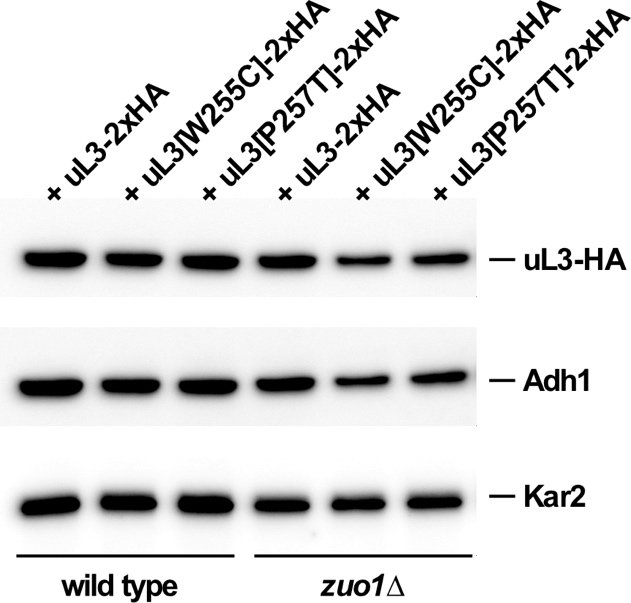
**

**FIGURE S5. Rodríguez-Galán *et al.***


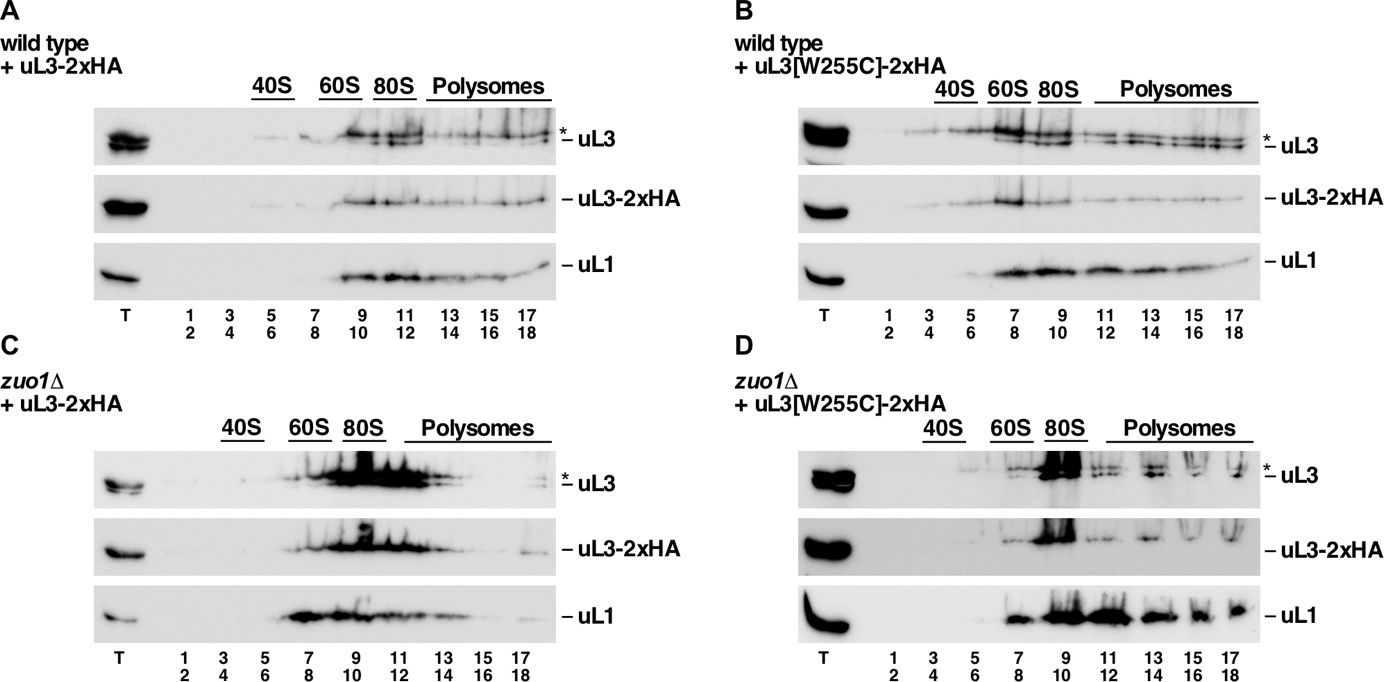


**FIGURE S6. Rodríguez-Galán *et al.***


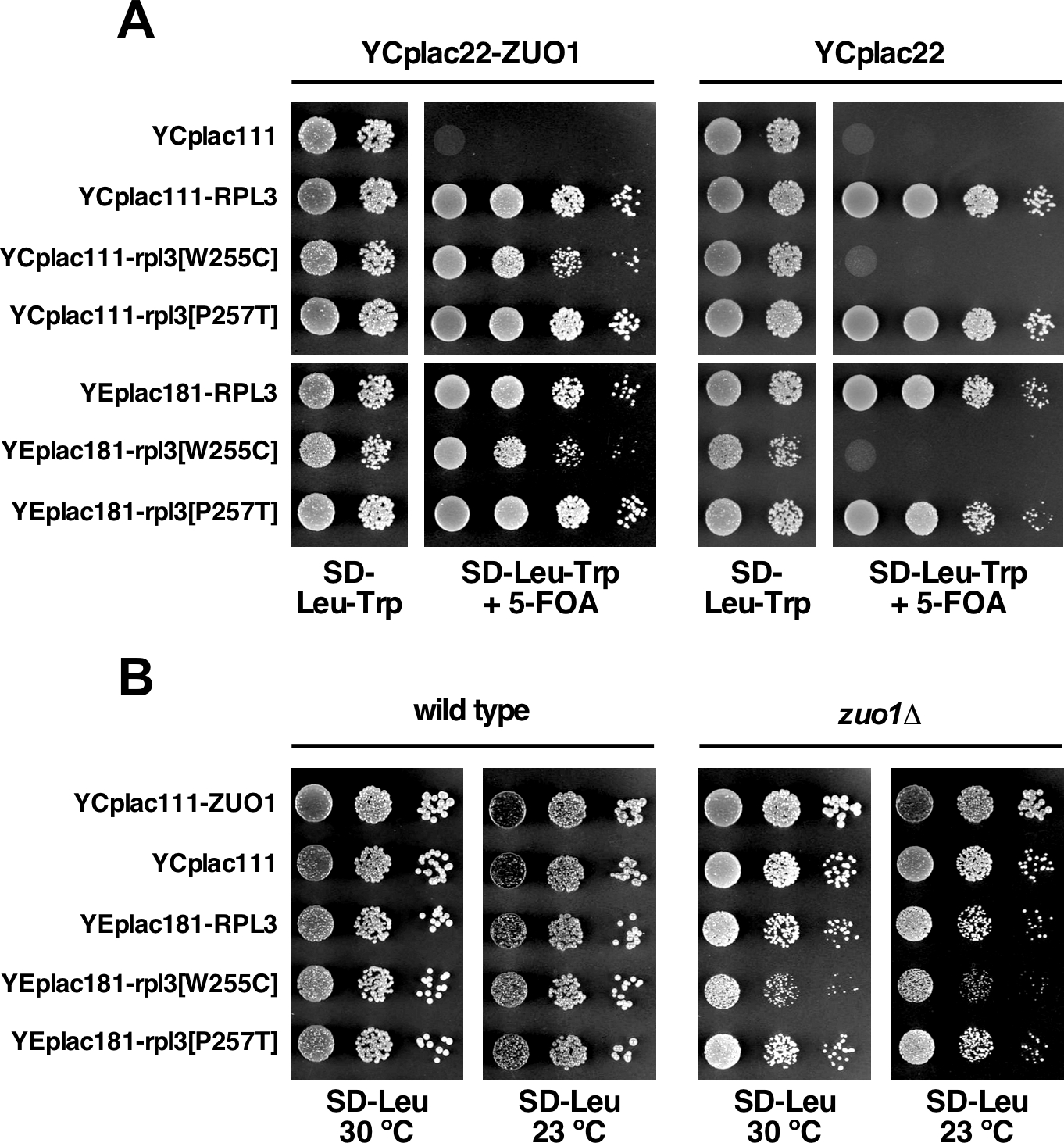


**FIGURE S7. Rodríguez-Galán *et al.***


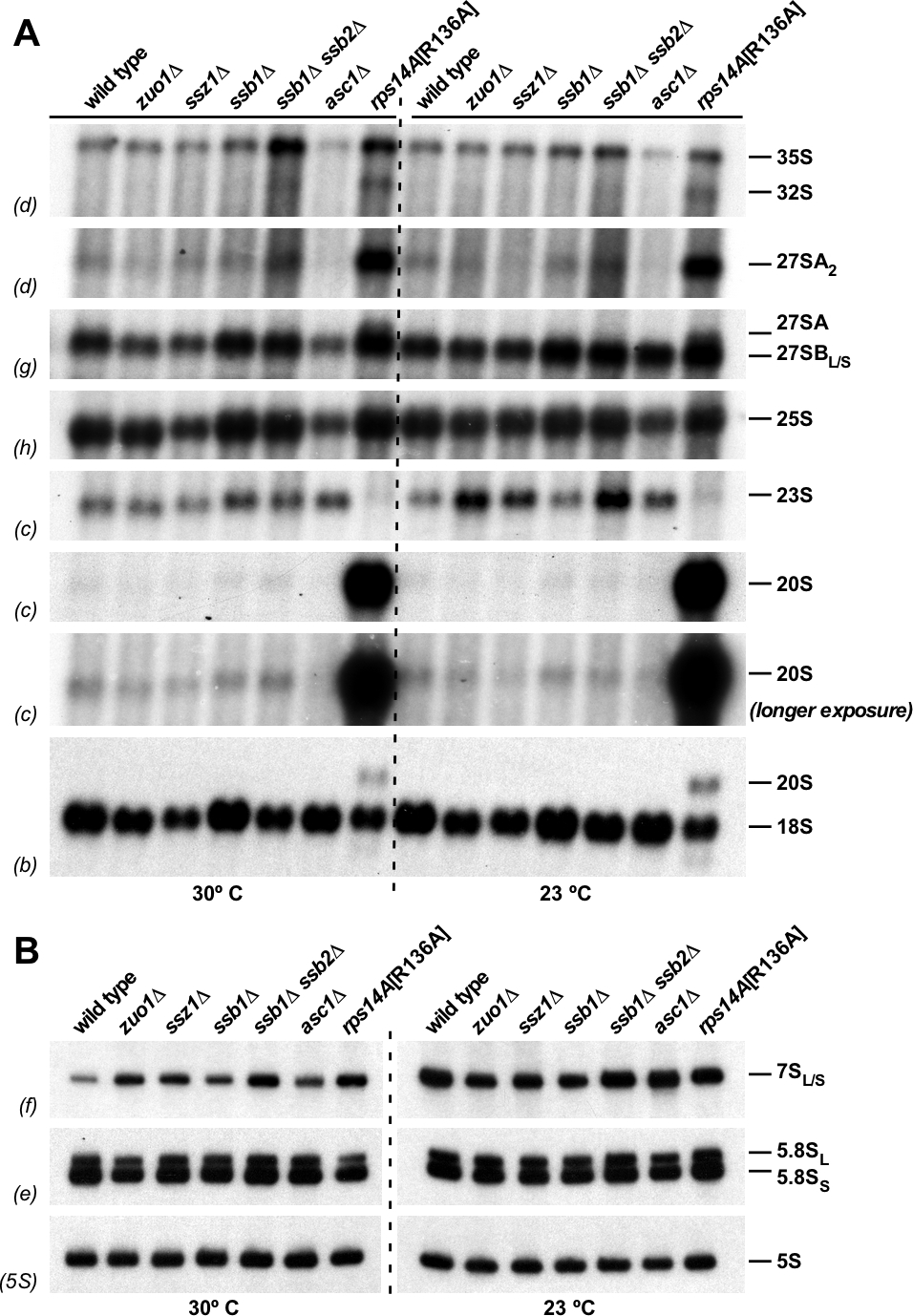


**FIGURE S8. Rodríguez-Galán *et al.***


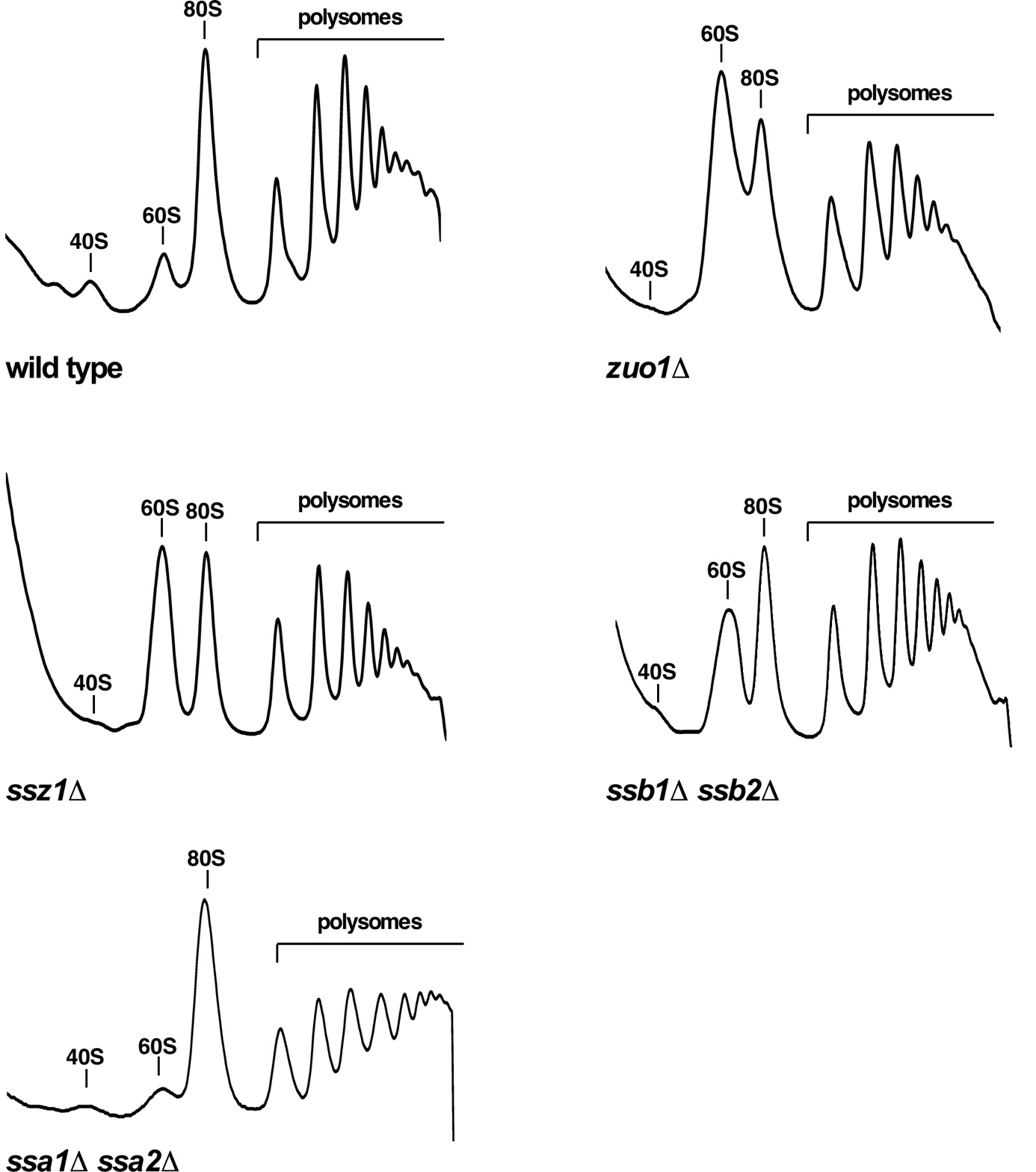


**FIGURE S9. Rodríguez-Galán *et al.***


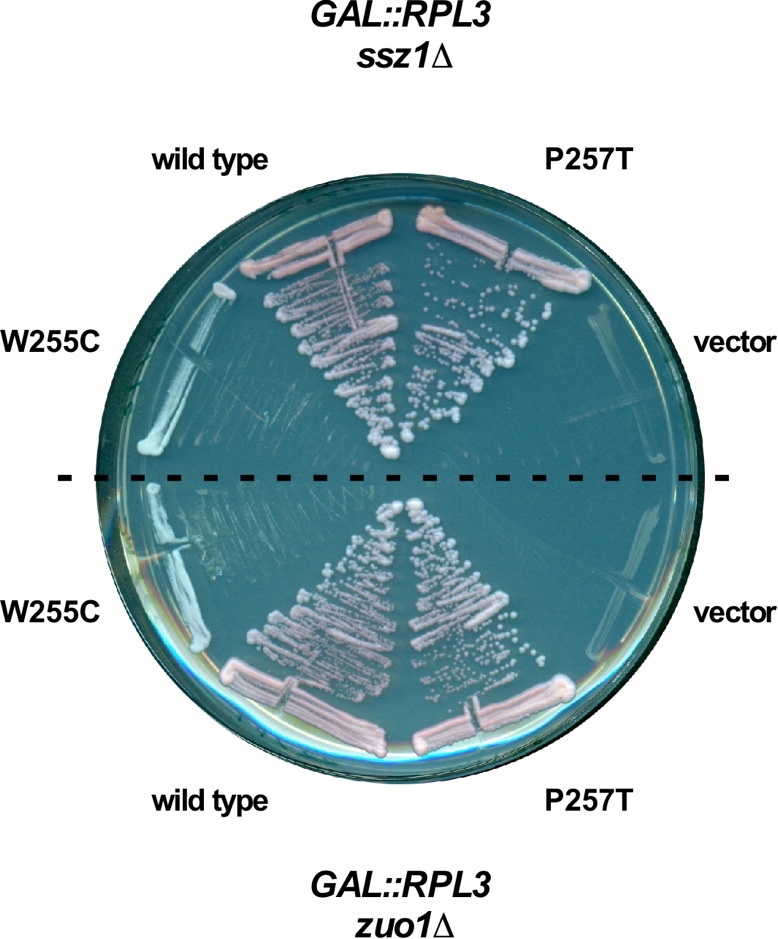


**FIGURE S10. Rodríguez-Galán *et al.***


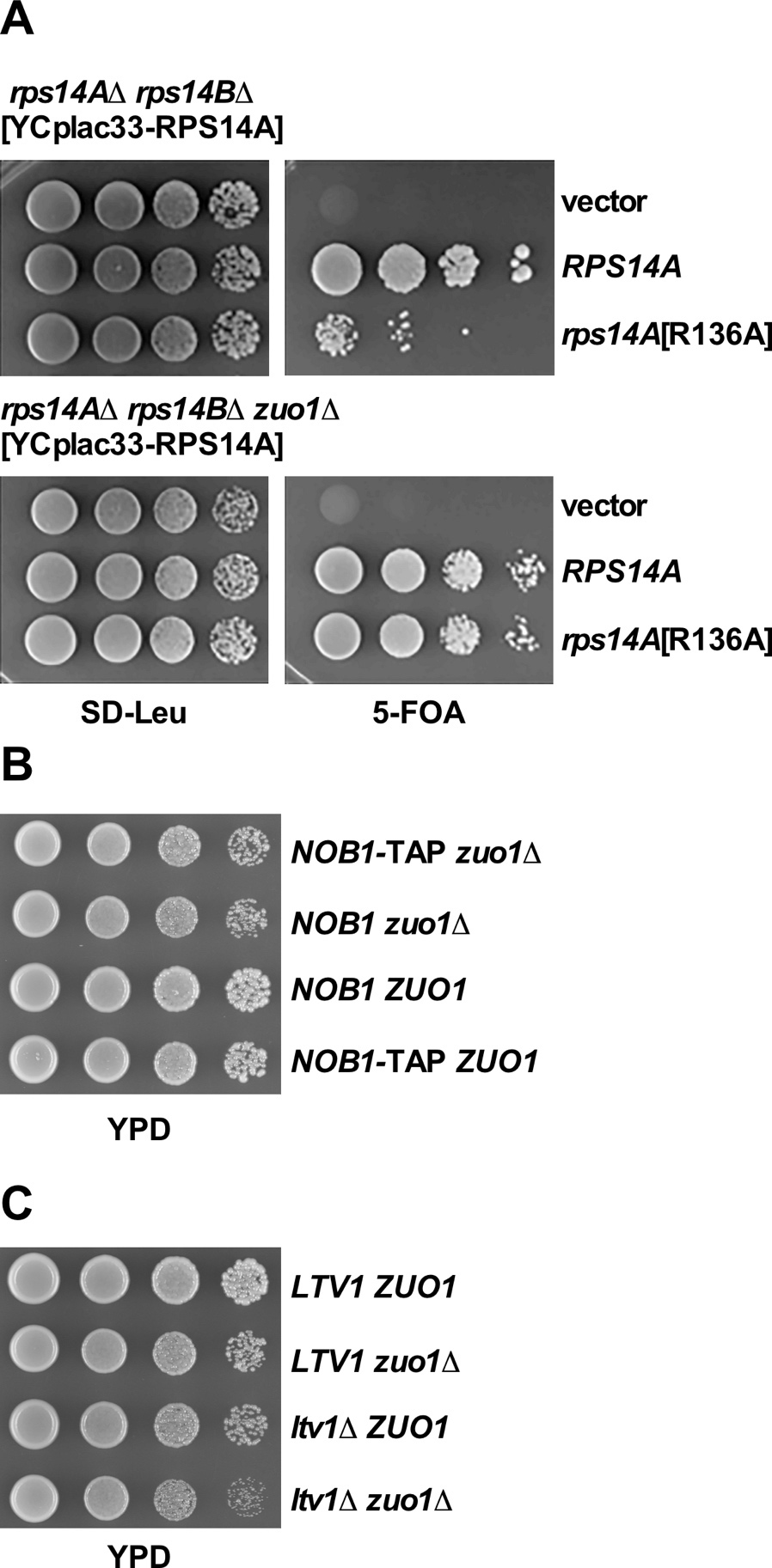


**FIGURE S11. Rodríguez-Galán *et al.***


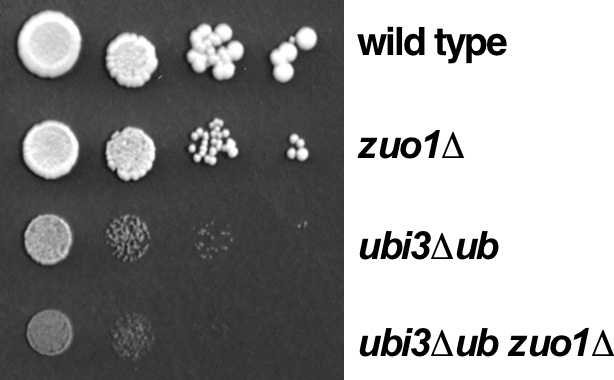


**FIGURE S12. Rodríguez-Galán *et al.***


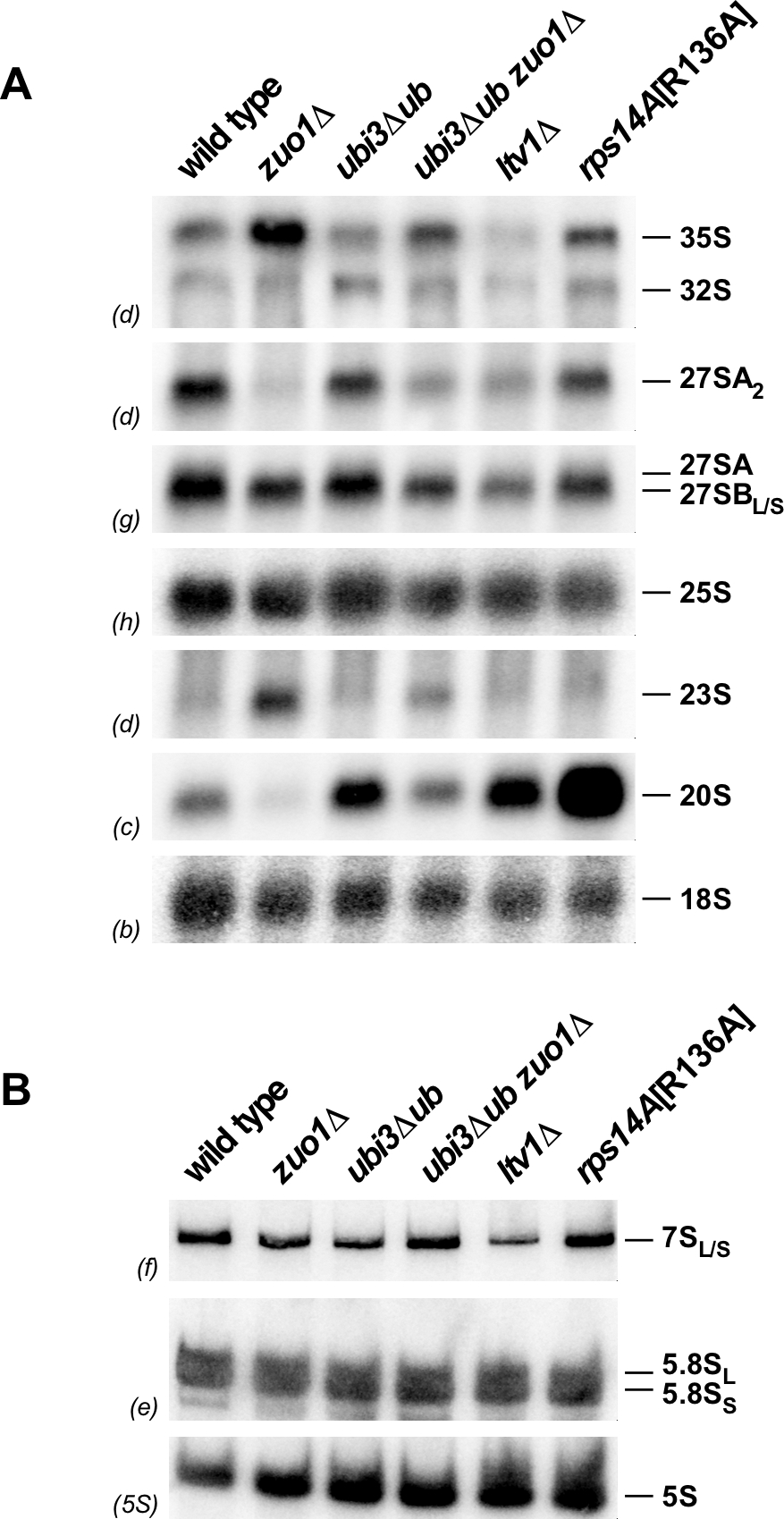


**FIGURE S13. Rodríguez-Galán *et al.***


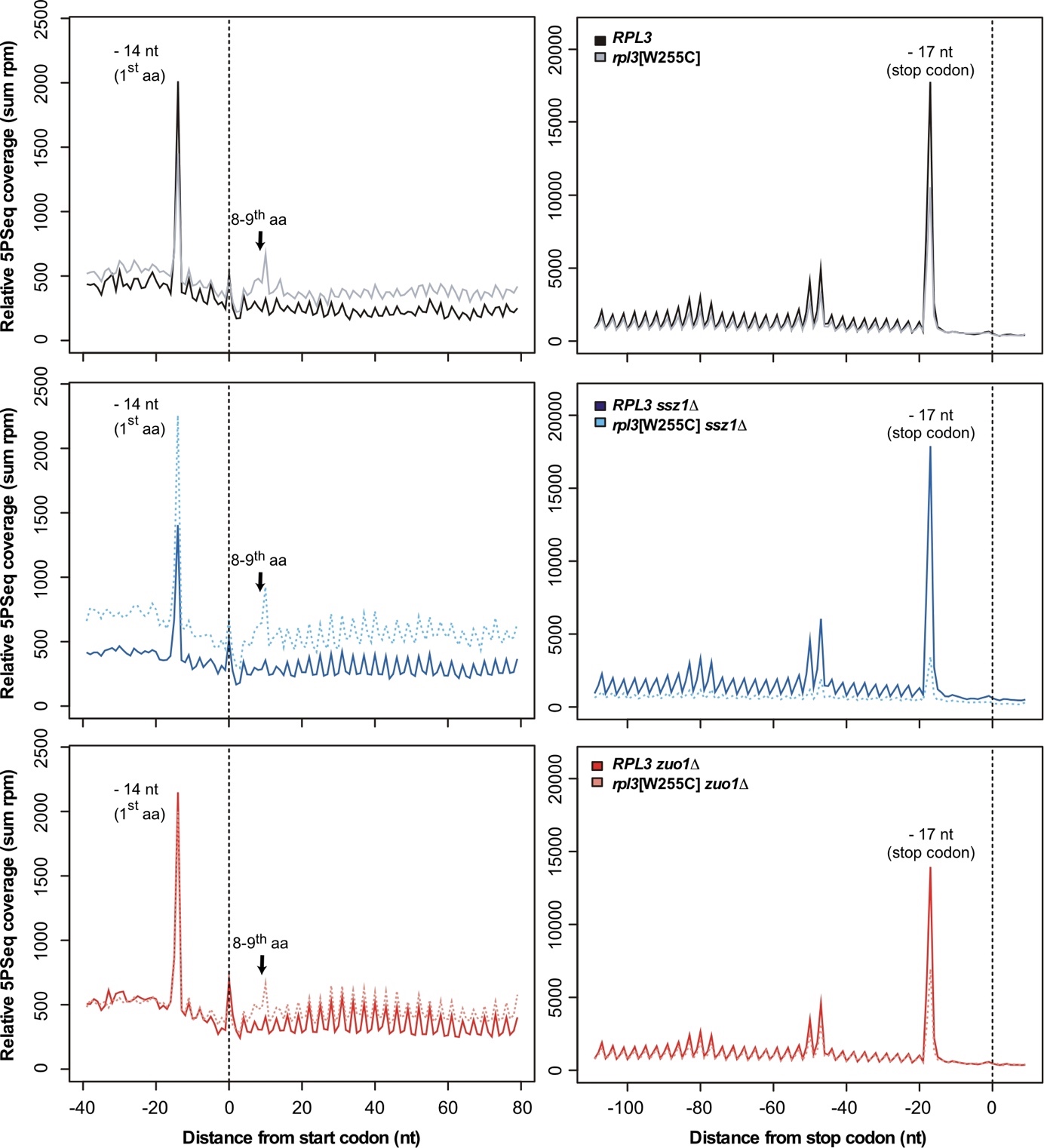


**FIGURE S14. Rodríguez-Galán *et al.***


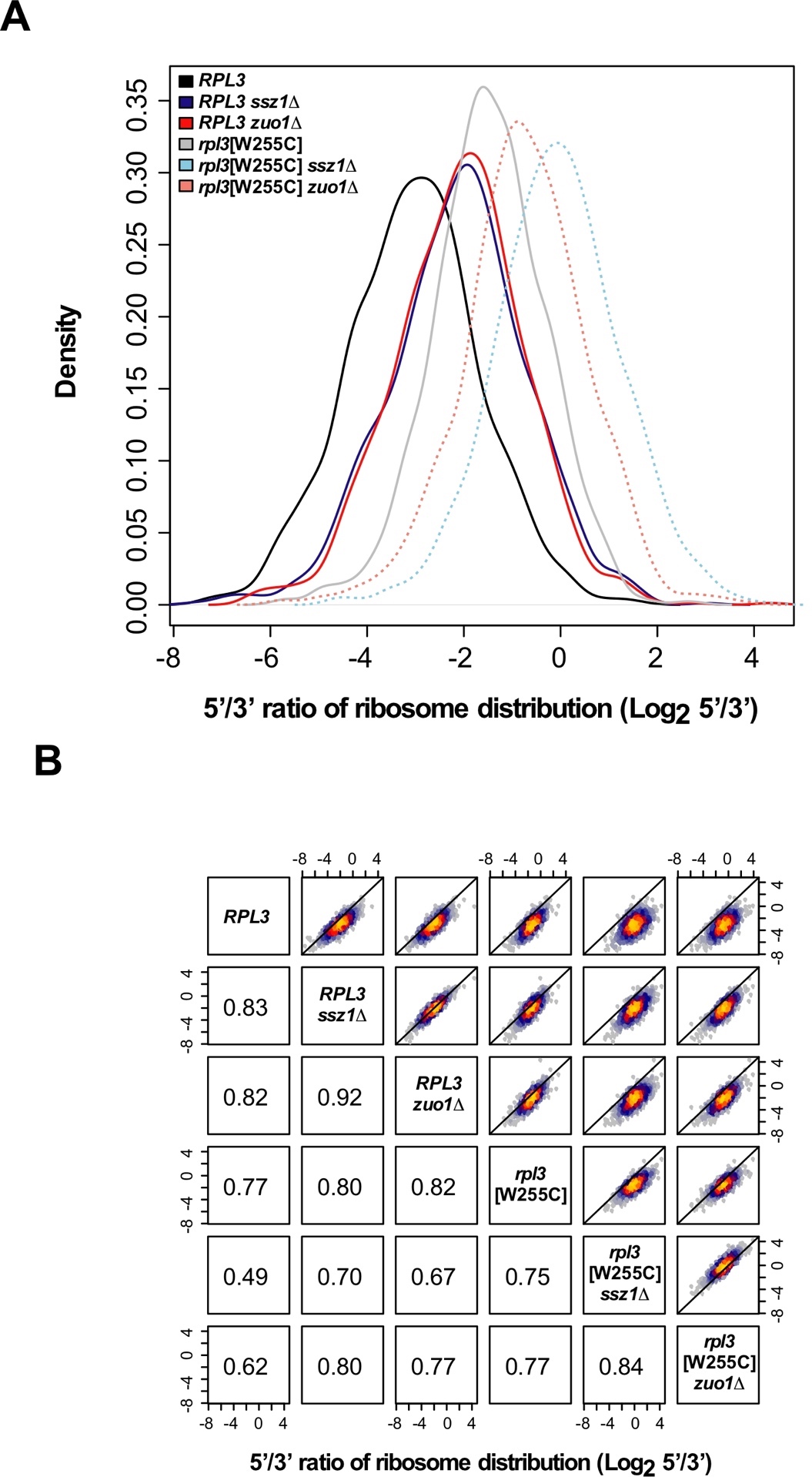


**FIGURE S15. Rodríguez-Galán *et al.***

**
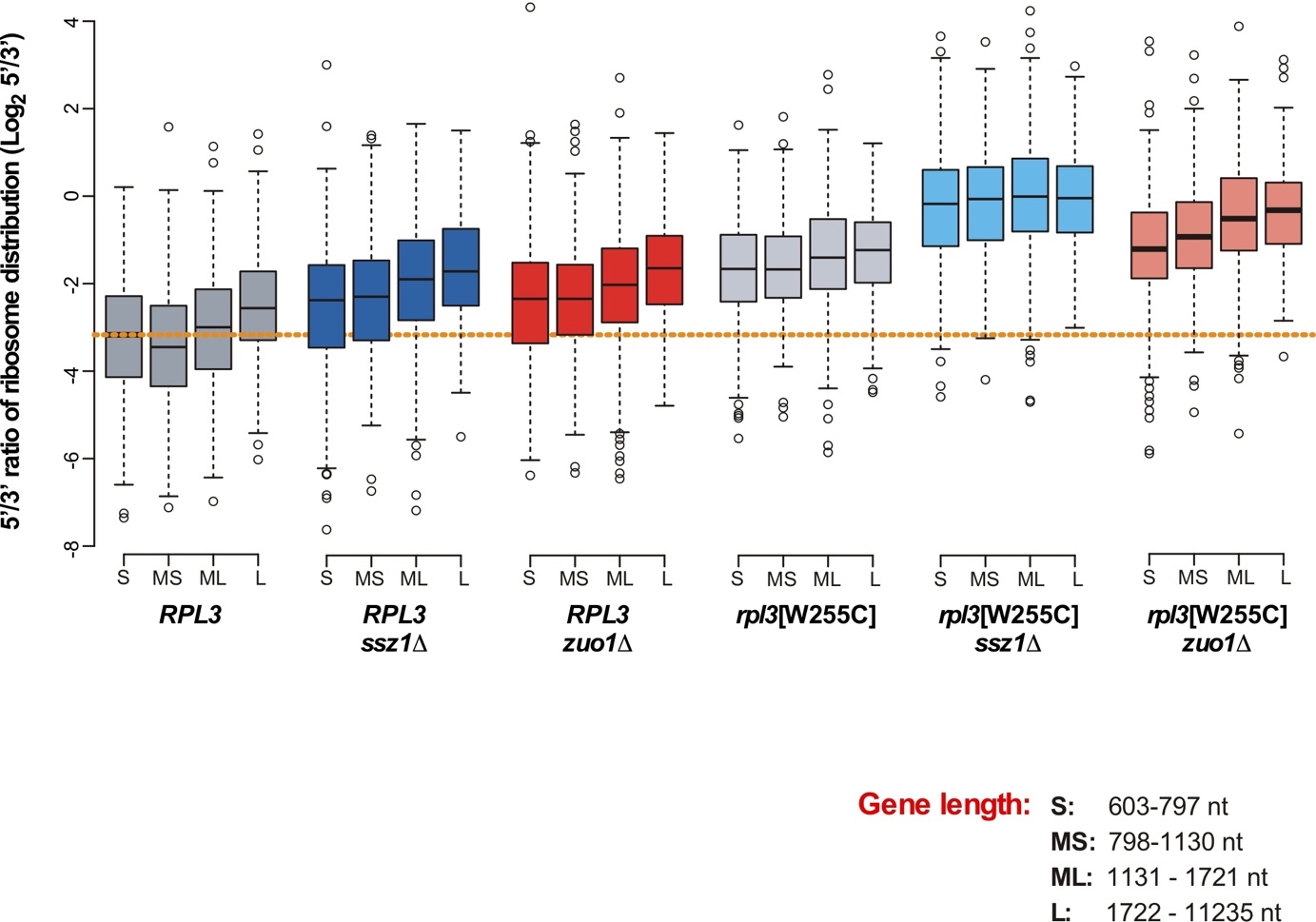
**

**FIGURE S16. Rodríguez-Galán *et al.***

**
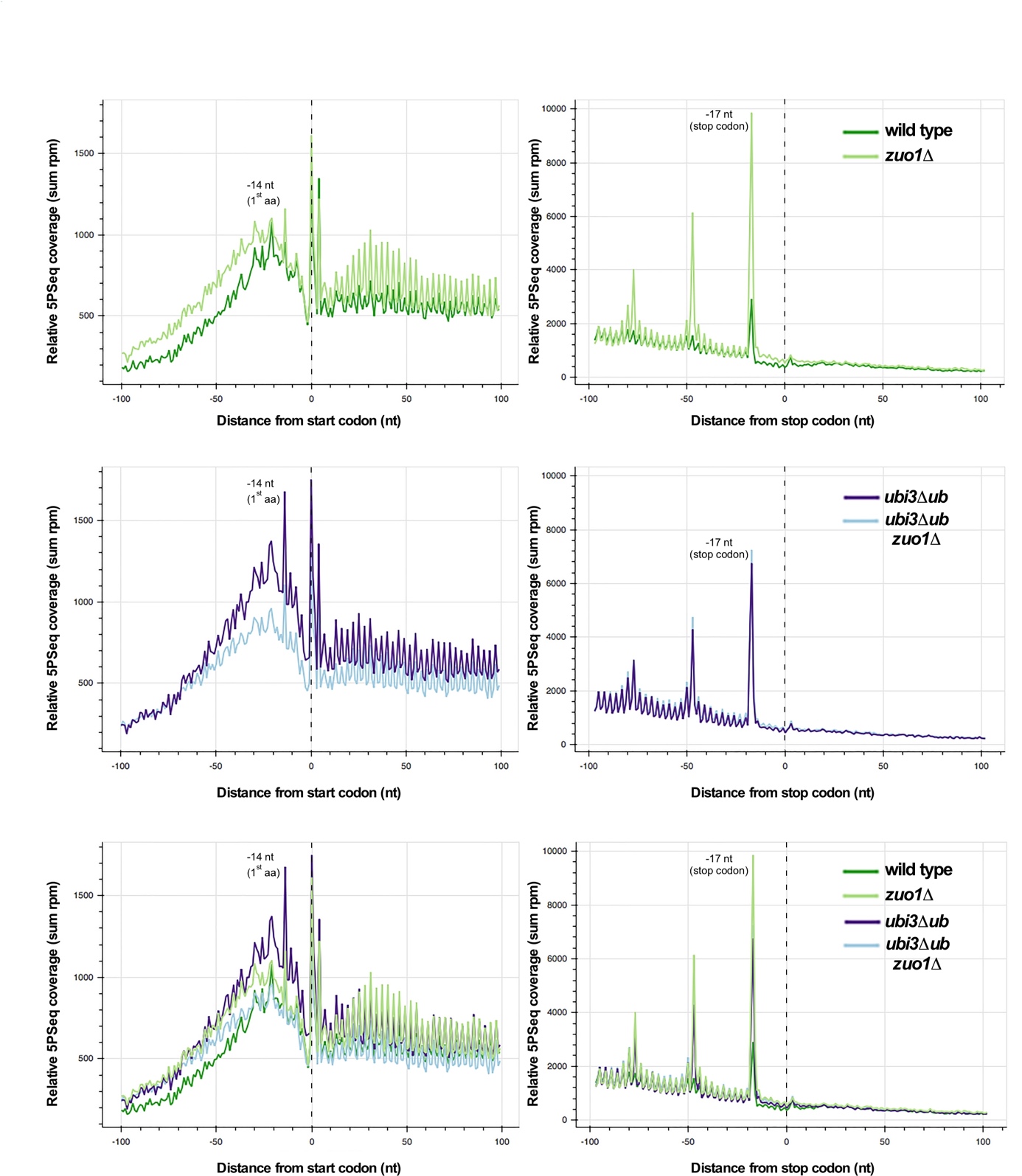
**

**FIGURE S17. Rodríguez-Galán *et al.***

**
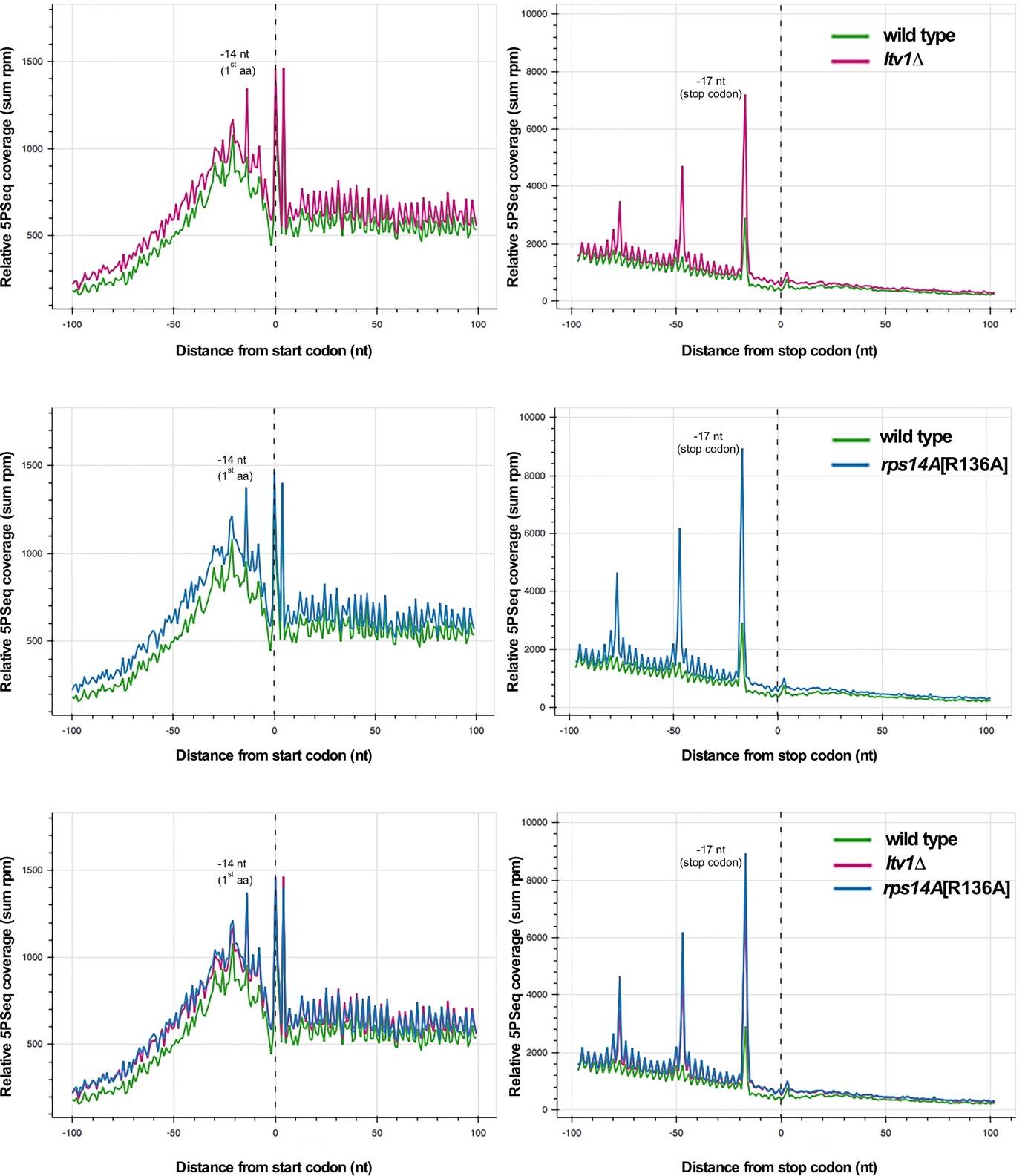
**

**FIGURE S18. Rodríguez-Galán *et al.***
